# Mapping axillary microbiota responsible for body odours using a culture-independent approach

**DOI:** 10.1186/s40168-014-0064-3

**Published:** 2015-01-24

**Authors:** Myriam Troccaz, Nadia Gaïa, Sabine Beccucci, Jacques Schrenzel, Isabelle Cayeux, Christian Starkenmann, Vladimir Lazarevic

**Affiliations:** Laboratory of Bacteriology, Geneva University Hospitals, Rue Gabrielle-Perret-Gentil 4, CH-1211 Geneva 14, Switzerland; Firmenich SA, Corporate R&D, Route des Jeunes 1, P.O. Box 148, CH-1211 Geneva 8, Switzerland; Genomic Research Laboratory, Geneva University Hospitals, Rue Gabrielle-Perret-Gentil 4, CH-1211 Geneva 14, Switzerland

**Keywords:** Skin, Bacterial communities, 16S rRNA gene sequencing

## Abstract

**Background:**

Human axillary odour is commonly attributed to the bacterial degradation of precursors in sweat secretions. To assess the role of bacterial communities in the formation of body odours, we used a culture-independent approach to study axillary skin microbiota and correlated these data with olfactory analysis.

**Results:**

Twenty-four Caucasian male and female volunteers and four assessors showed that the underarms of non-antiperspirant (non-AP) users have significantly higher global sweat odour intensities and harboured on average about 50 times more bacteria than those of AP users. Global sweat odour and odour descriptors sulfury-cat urine and acid-spicy generally increased from the morning to the afternoon sessions. Among non-AP users, male underarm odours were judged higher in intensity with higher fatty and acid-spicy odours and higher bacterial loads. Although the content of odour precursors in underarm secretions varied widely among individuals, males had a higher acid: sulfur precursor ratio than females did. No direct correlations were found between measured precursor concentration and sweat odours. High-throughput sequencing targeting the 16S rRNA genes of underarm bacteria collected from 11 non-AP users (six females and five males) confirmed the strong dominance of the phyla Firmicutes and Actinobacteria, with 96% of sequences assigned to the genera *Staphylococcus*, *Corynebacterium* and *Propionibacterium*. The proportion of several bacterial taxa showed significant variation between males and females. The genera *Anaerococcus* and *Peptoniphilus* and the operational taxonomic units (OTUs) from *Staphylococcus haemolyticus* and the genus *Corynebacterium* were more represented in males than in females. The genera *Corynebacterium* and *Propionibacterium* were correlated and anti-correlated, respectively, with body odours. Within the genus *Staphylococcus*, different OTUs were either positively or negatively correlated with axillary odour. The relative abundance of five OTUs (three assigned to *S. hominis* and one each to *Corynebacterium tuberculostearicum* and *Anaerococcus*) were positively correlated with at least one underarm olfactory descriptor.

**Conclusions:**

Positive and negative correlations between bacterial taxa found at the phylum, genus and OTU levels suggest the existence of mutualism and competition among skin bacteria. Such interactions, and the types and quantities of underarm bacteria, affect the formation of body odours. These findings open the possibility of developing new solutions for odour control.

**Electronic supplementary material:**

The online version of this article (doi:10.1186/s40168-014-0064-3) contains supplementary material, which is available to authorized users.

## Background

Human axillary odour is commonly attributed to the bacterial degradation of precursors in sweat secretions [1-3]. Age [4-6], sex [7-10], genetic factors [11-15], environmental factors (climate or stress situation) [1,16], hygiene and the use of cosmetics [6,17-19] may contribute to body odour by influencing the quantity and quality of secretions, or the types of bacteria present on skin. A culture-based approach used previously to isolate odour-generating bacteria was successful in identifying *Corynebacterium* and *Staphylococcus* species, in particular, *Corynebacterium striatum*, *C. jeikeium* and *Staphylococcus haemolyticus* as implicated in the generation of odourous volatiles [20-23].

The stability of the skin microbiota has been attributed to factors such as physiological skin pH, relative skin humidity, skin lipid composition, desquamation of the stratum corneum and skin temperature. A stable skin microbiota is involved in host resistance against skin pathogens [24]. In the underarms, distinct species may, however, be isolated in different individuals under certain conditions and may influence body odour formation. Two distinct types of axillary microbiota dominated by either coryneforms or cocci have been reported, the former being more prevalent in males and contributing to a more pronounced body odour [3,7]. Similarly, under conditions of high nutrient supply and humidity, the growth of coryneforms is favoured, and they may also be able to suppress the growth of cocci [2,7]. Women have 75% more apocrine glands in their armpits than men, but male apocrine glands are larger and may be more active in order to supply nutrients for bacterial growth [6].

Hygiene habits such as shaving axillae or use of cosmetics and antiperspirants (APs) may alter the odour profile by changing sweat volumes, the microbiota profile and its metabolic activity. Some cosmetics may contain nutrients such as glycerine, amino acids and hydrolysed collagen for the resident microbiota, or they may contain antimicrobials that increase the presence of resistant strains on skin [17].

Recent studies have demonstrated that some soluble odourless compounds are secreted by apocrine glands and some are unique to the human species [25]. These soluble compounds are transformed by the microbiota to release odorant carboxylic acids, sulfur compounds and odorant steroids [20-22,26].

To elucidate the correlations between axillary microbiota, body odours and the concentrations of acid and sulfur compound precursors, we performed a 3-day clinical study that included 24 subjects. The axillary bacterial load was quantified, and the axillary odour was evaluated for all subjects by four trained assessors. In addition, pyrosequencing of the 16S rDNA amplicon libraries of the axillary microbiome from 11 non-AP users allowed us to correlate bacterial taxa abundance with body odours.

## Results and discussion

### Subjects and sampling

Twenty-four subjects completed the study (Additional file 1: Table S1). They consisted of 13 AP users (seven females and six males) and 11 non-AP users (six females and five males). To assess axillary skin pH, odours and bacterial communities, the subjects participated in morning and afternoon sessions during the first 2 days. Sweat was collected at day 3 of the study.

### Physico-chemical measurements

Axillary pH was found to be significantly lower in AP users than in non-AP users for both females (median 4.6 vs. 6.3, respectively) and males (median 5.2 vs. 6.3, respectively) (Additional file 2: Figure S1 and Additional file 3: Table S2). In AP users, the median axillary pH was higher in males than in females at all sessions and increased between the morning and the afternoon sessions in both sexes.

In line with previous studies [27], the concentrations of odour precursors in sweat varied widely (Additional file 1: Table S1), and the average ratio between the acid precursor **1** (*N*-α-3-hydroxy-3-methylhexanoyl-(L)-glutamine) and the sulfur precursor **3** (*S*-[1-(2-hydroxyethyl)-1-methylbutyl]-(L)-cysteinylglycine) was about three times higher in males than it was in females (median 99 and mean 125 μg/mL vs. median 44 and mean 38 μg/mL, respectively). In addition, we found that the average and median ratios between the acid precursors **1** + **2** (*N*-α-3-methylhexenoyl-(L)-glutamine) and the sulfur precursors **3** + **4** (*S*-[1-(2-hydroxyethyl)-butyl]-(L)-cysteinylglycine) were about two times higher in males than those were in females in both AP and non-AP users (Additional file 4: Figure S2).

### Olfactory evaluation

The four trained assessors evaluated the left and right axillae of the subjects across four sessions. The intensities of each descriptor (from 0 to 10) were averaged for the four assessors. Only the five descriptors for which the intensities were rated higher than one for at least one subject were considered in the following analyses: **global** sweat (global), **sulfury-cat urine**-grapefruit-blackcurrant (sulfury-cat urine), **acid-spicy**-cumin (acid-spicy), **fatty**-foot-wet/mould sponge (fatty) and fresh onion. The means and ranges of the intensities of the odour descriptors across the four assessors are given in Additional file 5: Table S3.

**Figure 1:**
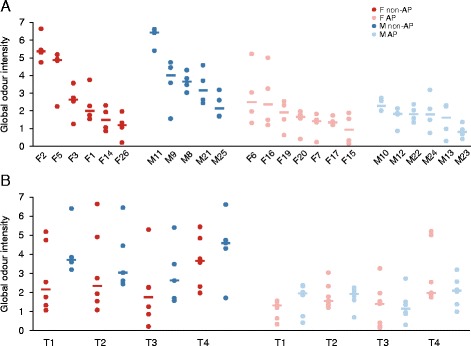
**Comparisons of underarm global sweat odour intensities.** Data for the left and right axillae were averaged for the four assessors (*circles*), and then, the median values (*thick*, *horizontal lines*) were calculated for **(A)** each subject or **(B)** each session (T1–T4) with respect to the sex and antiperspirant user type. Odour intensity was evaluated on a scale from 0 to 10. T1: Morning session on day 1; T2: Afternoon session on day 1; T3: Morning session on day 2; T4: Afternoon session on day 2; AP: Antiperspirant user; Non-AP: Non-antiperspirant user; F: Female; M: Male.

Global sweat odour was positively correlated with the other four descriptors (Spearman *r* > 0.61) (Additional file 1: Table S1), in particular with fresh onion (*r* = 0.765) and sulfury-cat urine descriptors (*r* = 0.810). No significant differences in odour intensity were noticed between the right and left axillae of the same subject (Additional file 1: Table S1 and Additional file 6: Figure S3). Significant differences in global odour were found between subjects from the non-AP user group (Kruskal-Wallis test *P* = 0.00042 for all individuals, *P* = 0.0066 for females only, *P* = 0.02 for males only), which was not the case in the AP user group (Figure 1A). Similar trends were observed with the other four odour descriptors.

The global sweat (Figure 1B), sulfury-cat urine and acid-spicy odours (Additional file 1: Table S1) generally increased from the morning to the afternoon sessions. Statistical significance of this effect was reached in several comparisons, including females only and participants of both sexes (Additional file 7: Table S4).

**Figure 2:**
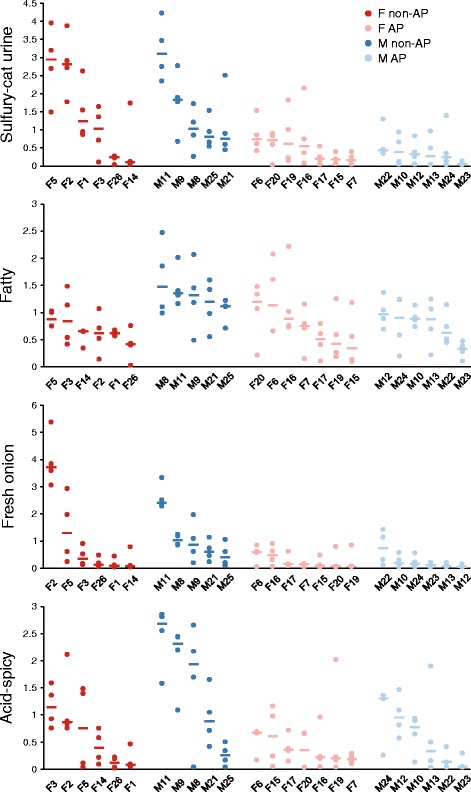
**Comparisons of underarm sweat odour intensities.** Data for the left and right axillae were averaged for the four assessors (*circles*), and then, the median values (*thick*, *horizontal lines*) were calculated for each subject. Odour intensity was evaluated on a scale from 0 to 10. AP: Antiperspirant user; Non-AP: Non-antiperspirant user; F: Female; M: Male.

Global sweat odour intensity was significantly higher in non-AP users (median 3.11) than in AP users (median 1.57) [Figure 1 and Additional file 3: Table S2]. Three other descriptors—sulfury-cat urine, acid-spicy and fresh onion—also tended to have higher intensities in non-AP users in both sexes, as was the case with the fatty descriptor in males (Figure 2). The underarms of non-AP males tended to have higher fatty and acid-spicy odour intensities in comparison with those of females (Figure 2). However, statistical significance was reached only for some of these observations (Additional file 3: Table S2). In most instances, statistically significant changes in the individual odour descriptors were associated with a statistically significant change in global odour (Additional file 3: Table S2 and Additional file 7: Table S4).

In non-AP females, we observed a trend of a lower axillary odour for the subjects over the age of 40 years and/or the subjects in the post-ovulation period. However, the significance of this observation remains to be clarified because of the small number of subjects in each subgroup.

### Analysis of axillary bacterial communities

#### Bacterial load

**Figure 3:**
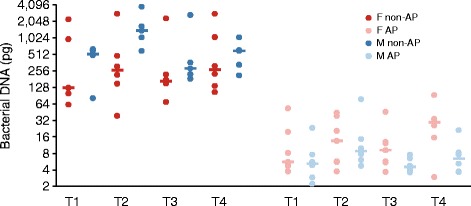
**Bacterial load on armpit skin as determined by qPCR in DNA extracts.** DNA quantity in purified extracts was calculated by using *S. aureus* MW2 genomic DNA as a reference. The *S. aureus* MW2 genome weighs approximately 2.9 fg and contains six 16S rDNA copies. The correlation between cycle threshold values and the *S. aureus* MW2 DNA amount in the qPCR mixture was linear in the 0.1 pg to 1 ng range. T1: Morning session on day 1; T2: Afternoon session on day 1; T3: Morning session on day 2; T4: Afternoon session on day 2; AP: Antiperspirant user; Non-AP: Non-antiperspirant user; F: Female; M: Male.

We used quantitative PCR (qPCR) with universal bacterial primers to assess bacterial loads on the armpit surface. Underarms of non-AP users harboured on average about 50 times more bacteria than did those of AP users (Figure 3). In AP and non-AP users of both sexes, the median bacterial concentration found in the afternoon was higher than that obtained in the morning samples. Among non-AP users, males had higher median bacterial loads than females did at each time point. The possibility of an inhibitory effect of DNA extracts from AP users on qPCR was excluded by comparing the cycle threshold value of the samples from selected AP users and non-AP users, either alone or mixed together (Additional file 8: Table S5).

#### 16S rDNA pyrosequencing

Because the armpit swabs from the AP users had very low DNA concentrations (Figure 3), amplification of the V1–3 region of the 16S rRNA gene by PCR using these samples did not produce a visible product in most instances. Pyrosequencing of the amplicon libraries derived from 44 samples of non-AP users and 11 (of 52) samples of AP users generated 423,308 raw reads. Therefore, we explored the diversity of skin microbiota of non-AP users only. A total of 237,947 pyrosequencing reads derived from the skin samples of non-AP users passed the quality control steps.

#### Putative contaminant sequences

To assess possible contamination of our samples by exogenous DNA, we sequenced 16S rDNA amplicons from the four negative controls (in a separate pyrosequencing run). A total of 825 quality-filtered sequence reads obtained for control samples were represented by 104 operational taxonomic units (OTUs) (Additional file 9: Table S6). Of these, 95 had a mean relative abundance more than 4.8-fold greater in controls than in samples. Two low-abundance OTUs had comparable abundance in the control and sample data sets (0.07% vs. 0.06% and 0.29% vs. 0.28%, respectively). Therefore, from the sample data set initially represented by 521 OTUs, we removed 97 putative contaminant OTUs. These OTUs corresponded to 86% and 2% of the total number of reads in the control data set and (non-decontaminated) sample data set, respectively. However, we cannot exclude that some of the sequences considered as contaminants derived from the bacteria genuinely present in the skin samples. Proteobacteria, known to be common water and reagent contaminants [28,29], were the most abundant phylum among putative contaminant OTUs, corresponding to 65% of sequence reads in the control data set. The proportion of putative contaminant 16S sequences in the (non-decontaminated) sample data set was clearly inversely correlated with the bacterial DNA concentration in purified DNA extracts (Spearman *r* = −0.874) (Additional file 10: Figure S4). A highly abundant OTU (OTU368907) assigned to *Propionibacterium acnes* had a similar proportion in the control and sample data sets (12.2% vs. 12.1%). We did not consider it as a contaminant because (i) it was found in high proportion in several samples with relatively high DNA concentration, and (ii) its relative abundance showed a subject-specific pattern. The six OTUs that had lower proportions in the control than in the sample data set, corresponding to 1.3% and 68.5% of total reads, respectively, were not removed from the sample data set.

#### Taxa abundance

After removal of putative contaminant 16S sequences, the number of sequences was normalized to the smallest number of sequences (995) found in any sample. The normalized data set was analysed at the phylum, genus and OTU levels.

**Figure 4:**
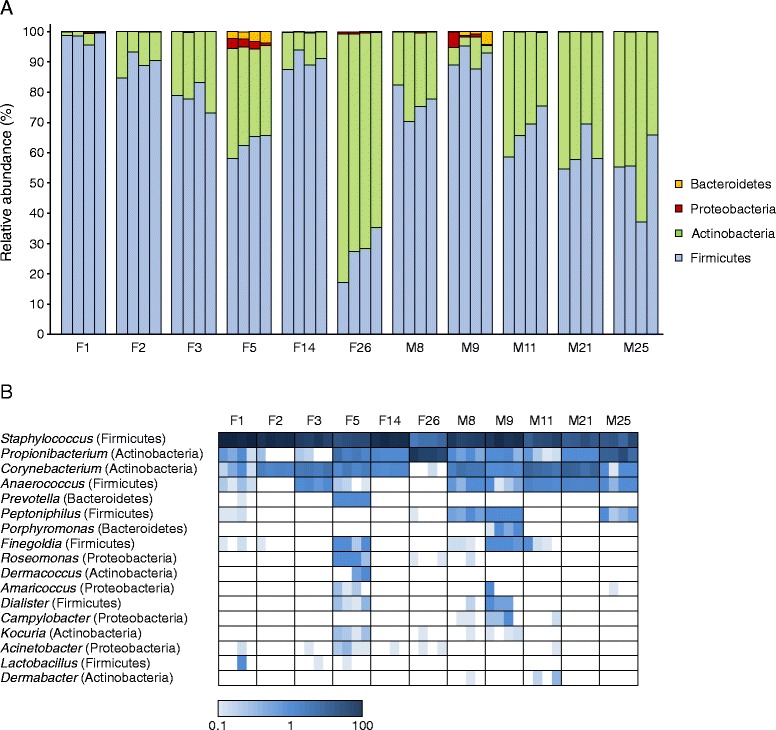
**Relative abundance of predominant bacterial taxa across skin microbiomes. (A)** Proportion of 16S sequences assigned to the four major phyla. For each subject, the four sessions (1–4) are presented in the left-to-right direction. **(B)** Heat map showing the relative abundance of genera across samples. Genera with a relative abundance >0.5% in at least one sample are presented. The four fields within a frame correspond to the four sessions (1–4) in the left-to-right direction. The proportion is indicated by the scale at the bottom of the plot. The analysed subjects were non-antiperspirant users.

Samples were greatly dominated by the phyla Firmicutes and Actinobacteria, followed by Proteobacteria and Bacteroidetes (Figure 4A). These four major phyla contributed together to over 99.8% of sequence reads in all samples. Members of other phyla (TM7, Planctomycetes, Cyanobacteria and Thermi) were found in few samples. The relative abundance of the two major phyla in the human skin microbiome, Firmicutes and Actinobacteria, has been shown to vary according to the topographical region [30,31]. The proportion of these two phyla in our study showed a high degree of congruence with the published data relative to the armpit skin microbiome (72.2% vs. 72.4% for Firmicutes and 26.8% vs. 27.2% for Actinobacteria) [31].

A total of 68 genera were identified in the data set. The genera *Staphylococcus* (Firmicutes), *Propionibacterium* (Actinobacteria) and *Corynebacterium* (Actinobacteria) constituted on average 96% (range 84.7%–100%) of the total 16S sequences (Figure 4B). Previous culture-based [2,3] and culture-independent [30,32] studies showed the dominance of these three genera in the axillary skin microbiota. In line with the data from Egert et al. [33], *Anaerococcus* and *Peptoniphilus* were the fourth and fifth most abundant genera in our study, respectively. Most other genera were present in low proportions; 49 of them occurred at a frequency lower than 0.11% across all samples.

Of 183 OTUs identified in the normalized data set, between 7 and 37 were present in individual samples (Additional file 11: Table S7). We addressed the question of whether there was a core microbiome, that is, ‘species-level’ OTUs present in all individuals investigated. One such OTU (OTU356733), belonging to *S. epidermidis*, was present in samples from all sessions. On average, it constituted 38.7% (range 2.8%–83.8%) of sequence reads. When the sequences corresponding to the four time samples of the same individual were combined into a single data set, three additional OTUs assigned to *S. hominis* (OTU154509), *C. tuberculostearicum* (OTU470219) and *P. acnes* (OTU368907) were found to be shared by all individuals. The proportion of sequence reads corresponding to such a defined universal core reached 81.5% (range 66.3%–98.7%). Therefore, our results show that the armpit microbiome has a relatively low OTU richness and that most 16S sequences belonged to the OTUs identified across all 11 individuals investigated. Low OTU richness and inter-personal variations were previously documented in skin microbiomes of the inner elbow, which is, like the armpit, a moist body site [34]. In contrast, microbiota from the palm surfaces, considered a dry site, were shown to contain >150 OTUs per subject and varied significantly not only between individuals but also within symmetrical sites within the same individual [35]. Of course, the results obtained in different studies are influenced by the respective experimental procedures and data analysis procedures.

#### Skin microbiota variations by subject, sex and time of day

**Figure 5:**
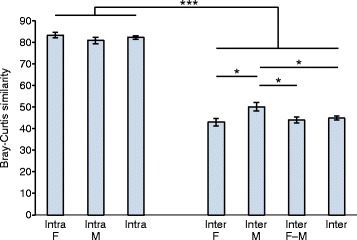
**Mean intra-individual and inter-individual similarity.** The Bray-Curtis similarity was calculated by using the square-root-transformed relative abundance of OTUs. To calculate inter-individual similarity, we compared samples taken at the same session. *Error bars* correspond to standard errors. *P < 0.05; ***P < 0.001 by two-sided *t* test; F: Female; M: Male; F-M: Comparison between males and females; Intra: Intra-individual; Inter: Inter-individual. The analysed subjects were non-antiperspirant users.

To assess microbiota variations among and within subjects, we constructed a Bray-Curtis similarity matrix on the basis of the square-root-transformed relative abundance of OTUs. Intra-individual variation of the skin microbiota over a 30-h period was smaller than inter-individual variation in both sexes (Figure 5).

**Figure 6:**
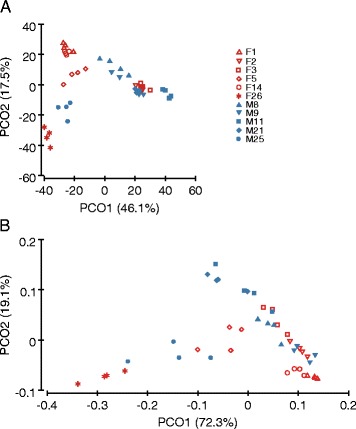
**Principal coordinate analysis for skin bacterial communities.** The analyses were performed by using **(A)** the Bray-Curtis similarity matrix based on the square-root-transformed relative abundance of OTUs and **(B)** weighted UniFrac based on the OTU relative abundance. The percentage of variation explained is given in parentheses. The analysed subjects were non-antiperspirant users.

A principal coordinate analysis of Bray-Curtis similarity showed that each individual carried a relatively specific bacterial community (Figure 6A). Clustering by subject was also observed when the phylogenetic distance between OTUs was taken into account using UniFrac [36] (Figure 6B). In line with these observations, several taxa were consistently found in a limited number of individuals. For instance, OTU268540 belonging to *Porphyromonas* and OTU381339 assigned to *C. simulans* were identified across the four time samples of the subject M9 and had a median abundance of 1.6% and 1.2%, respectively, but they were not found in any other subjects. Similarly, two *Staphylococcus* OTUs (OTU107105 and OTU244892) were found only in subject M25 (median abundance 0.85% and 0.95%, respectively). Another OTU from *Staphylococcus* (OTU330679) was found in four individuals at all time points, with a median relative abundance in the 0.55%–14.5% range, but was not found in samples from subject F26.

A permutational multivariate ANOVA (PERMANOVA) test based on Bray-Curtis similarity or UniFrac distance matrices did not reveal significant differences in the overall microbiota structure between sexes at any time point (not presented). Although global analysis did not reveal clear clustering by sex (Figure 6), the proportion of several bacterial taxa showed significant variation between males and females. The genera *Anaerococcus* and *Peptoniphilus* were less represented in females than in males (Table 1). Similarly, the abundance of an *Anaerococcus* OTU (OTU14290) was significantly higher among males. This OTU was found in all (20) samples from male subjects, with a median proportion (per subject) in the 0.4%–3.17% range. In contrast, only seven (of 24) samples from females had detectable levels of OTU14290, the proportion of which was 0.1%–0.2%. Five other OTUs had higher prevalence and relative abundance in males than in females, and these differences reached statistical significance for at least one time point. Three of these OTUs belonged to the genus *Corynebacterium* (OTU13430, OTU416589 and OTU494493), the others being *Peptoniphilus asaccharolyticus* (OTU489717) and *S. haemolyticus* (OTU354779) (Table 1).

**Table 1:** **Statistically significant differences in the proportion of genera and OTUs between sexes**

**Genus or OTU**	**Median F**	**Median M**	**Session**	***P*** ^**a**^	**F vs. M (%)** ^**b**^
*Anaerococcus*	0.20	3.72	T1	**0.021**	−95
	0.05	0.60	T2	0.096	−92
	0	1.11	T3	**0.019**	−100
	0.05	1.21	T4	0.053	−96
*Peptoniphilus*	0	0.80	T1	0.19	−100
	0	0.40	T2	0.11	−100
	0	0.60	T3	0.11	−100
	0	0.80	T4	**0.048**	−100
*Corynebacterium* OTU13430	0	0	T1	0.14	–
	0	0	T2	0.35	–
	0	0	T3	0.35	–
	0	0.10	T4	**0.044**	−100
*Corynebacterium* OTU494493	0	0.10	T1	**0.048**	−100
	0	0	T2	0.14	–
	0	0	T3	0.14	–
	0	0	T4	0.35	–
*Staphylococcus haemolyticus* OTU354779	0	0	T1	0.14	–
	0	0	T2	0.14	–
	0	0.40	T3	**0.048**	−100
	0	0	T4	0.14	–
*Anaerococcus* OTU14290	0	1.21	T1	**0.0055**	−100
	0.05	0.50	T2	**0.014**	−90
	0	0.60	T3	**0.0066**	−100
	0	0.70	T4	**0.0054**	−100
*Corynebacterium* OTU416589	0	0.30	T1	0.11	−100
	0	0.10	T2	**0.048**	−100
	0	0.30	T3	**0.015**	−100
	0	0	T4	0.14	–
*Peptoniphilus asaccharolyticus* OTU489717	0	0.80	T1	0.11	−100
	0	0.40	T2	**0.048**	−100
	0	0.60	T3	0.11	−100
	0	0.80	T4	**0.048**	−100

*F* female, *M* male, *T1* morning session on day 1, *T2* afternoon session on day 1, *T3* morning session on day 2, *T4* afternoon session on day 2, −, the median is 0 in both groups whereas the mean is lower in females than in males.

^a^ *P* value determined by the Mann-Whitney *U* test. *P* values are reported for all sampling points whenever the difference between the groups compared was statistically significant in at least one time point. Significant *P* values (<0.05) are given in bold.

^b^Change in median value in female-to-male comparison.

The higher abundance of the genera *Anaerococcus* and *Corynebacterium* in males than in females has also been observed in a study of upper buttock skin bacterial communities [31]. Similarly, *Corynebacterium* was reported to be more abundant on hand surfaces of males. However, differences between male and female microbiomes seem to be driven by different taxa at various anatomical sites. For instance, when female skin microbiomes were compared with those of males, *Propionibacterium* was enriched on the upper buttock skin [31] but reduced on the palm surface [35].

Both the median and mean values of OTU richness and of the Shannon diversity index showed a tendency to increase in males relative to females, as well as to slightly decrease in the afternoon compared with the morning samples (Additional file 12: Figure S5). However, these differences did not reach the level of statistical significance (Mann-Whitney *U* test) at any given session (for sex comparison) or on any given day (for morning-to-afternoon comparison). In previous studies, inter-sex differences in bacterial diversity varied between anatomical sites; females showed higher diversity of the skin microbiome at the palm [35], forearm and forehead (subjects using make-up) surfaces [37] but lower diversity on the upper buttock skin [31].

### Correlation between bacterial taxa

To assess correlations between different members of the skin microbiome, as well as between body odours and specific bacterial taxa, we used the Spearman rank test. Both positive and negative correlations at the phylum, genus and OTU levels were identified, suggesting the existence of mutualism and competition among the skin bacteria. At the phylum level, the strongest correlation was found between Actinobacteria and Firmicutes (*r* = −0.991), because they predominated in all samples. The relative abundance of the genus *Propionibacterium* (median 1.8%, mean 13.5%) was inversely correlated with those of *Corynebacterium* (median 7.4%, mean 13.2%) and *Staphylococcus* (median 72.3%, mean 69.4%). Several genera with low relative abundance showed positive correlations between each other (Additional file 13: Table S8).

Strong positive correlations were found among the OTUs assigned to the genus *Corynebacterium* (Additional file 13: Table S8). Within the genus *Staphylococcus*, different OTUs were either positively or negatively correlated. For instance, the two most abundant staphylococcal OTUs (median relative abundance of 32% and 13% for *S. epidermidis* OTU356733 and *S. hominis* OTU154509, respectively) tended to exclude each other (*r* = −0.703).

### Taxon-odour correlations

In our study, individual variations in odour precursor concentration did not correlate with the intensity value of sweat odours (and odour descriptors) (−0.5 < Spearman *r* < 0.5). In contrast, the relative abundance of six OTUs (three from *S. hominis* and one each from *C. tuberculostearicum*, *Corynebacterium* and *Anaerococcus*) was positively correlated (*r* > 0.5) with at least one olfactory descriptor (Table 2). *P. acnes* OTU368907 was negatively correlated with odours. Some of the correlations between OTUs and body odours were reflected at the genus level: the genera *Corynebacterium* and *Propionibacterium* were positively correlated and anti-correlated, respectively, with global sweat odour intensity.

**Table 2:** **Correlation between the relative abundance of bacterial taxa (operational taxonomic units [OTUs] or genera) and odour intensity in the right axilla**

**Odour**	**Taxon**				**Spearman** ***r*** ^**b**^
	**Phylum; genus or species; OTU ID**	**Relative abundance**		**Positive samples** ^**a**^	
		**Mean**	**Median**		
Global	Actinobacteria; *Corynebacterium tuberculostearicum*; OTU470219	8.74	5.83	41	0.586
Fresh onion	Actinobacteria; *Corynebacterium tuberculostearicum*; OTU470219	8.74	5.83	41	0.515
Global	Actinobacteria; *Corynebacterium*; OTU13430	0.44	0.00	13	0.547
Global	Firmicutes; *Staphylococcus hominis*; OTU154509	21.51	13.47	42	0.605
Fresh onion	Firmicutes; *Staphylococcus hominis*; OTU154509	21.51	13.47	42	0.648
Spicy	Firmicutes; *Staphylococcus hominis*; OTU154509	21.51	13.47	42	0.659
Global	Firmicutes; *Staphylococcus hominis*; OTU173469	2.64	1.36	33	0.571
Fresh onion	Firmicutes; *Staphylococcus hominis*; OTU173469	2.64	1.36	33	0.603
Acid-spicy	Firmicutes; *Staphylococcus hominis*; OTU173469	2.64	1.36	33	0.671
Acid-spicy	Firmicutes; *Staphylococcus hominis*; OTU338191	0.18	0.10	25	0.659
Fresh onion	Firmicutes; *Staphylococcus hominis*; OTU338191	0.18	0.10	25	0.509
Fatty	Firmicutes; *Anaerococcus*; OTU14290	0.54	0.10	27	0.600
Global	Actinobacteria; *Propionibacterium acnes*; OTU368907	12.49	0.55	35	−0.538
Acid-spicy	Actinobacteria; *Propionibacterium acnes*; OTU368907	12.49	0.55	35	−0.588
Global	Actinobacteria; *Corynebacterium*	13.16	7.39	41	0.540
Acid-spicy	Actinobacteria; *Corynebacterium*	13.16	7.39	41	0.537
Global	Actinobacteria; *Propionibacterium*	13.46	1.76	39	−0.513
Acid-spicy	Actinobacteria; *Propionibacterium*	13.46	1.76	39	−0.557

^a^Number of samples in which the given taxon was detected (total samples = 44).

^b^Only correlations with Spearman *r* > 0.5 or < −0.5 are presented.

Our results are in agreement with previous culture-based assessments of the axillary bacteria-odour relation, which showed that certain members of the genus *Staphylococcus* were involved in the generation of odours [2]. We also confirm the observation reported by Taylor et al*.* [3] that, at the genus level, *Staphylococcus* does not correlate with body odours. Indeed, both the most abundant OTU (OTU356733) and a highly abundant OTU (OTU330679) from the species *S. epidermidis* (Additional file 13: Table S8) were weakly anti-correlated with body odours (not presented), whereas *S. hominis* OTUs were positively associated with odours (Table 2). Our observation that the genus *Corynebacterium* correlates with body odours is also in agreement with the data from a culture-based study [3]. James et al*.* [1] reported that 16S rRNA gene sequencing of the predominant colony type of aerobic axillary corynebacteria had the best sequence match to *C. tuberculostearicum*. Other corynebacteria such as *C. striatum* [21] and *C. jeikeium* [38,39] have been reported as odour-generating microorganisms in the underarms. Our data indicate that the most abundant OTU (OTU470219) from the genus *Corynebacterium* is assigned to *C. tuberculostearicum*.

**Figure 7:**
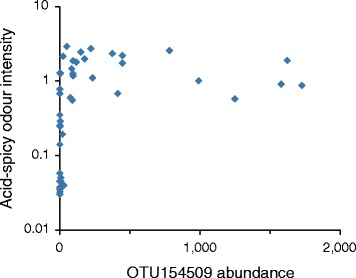
**Acid-spicy odour intensity as a function of the ‘absolute’ abundance of OTU154509.** Odour intensity was evaluated on a scale from 0 to 10. The ‘absolute’ abundance is expressed in arbitrary units obtained by multiplying the relative abundance of the OTUs (fraction of all OTUs determined by pyrosequencing) with DNA quantity in pg determined by qPCR in purified extracts using *S. aureus* MW2 genomic DNA as a reference. The *S. aureus* MW2 genome weighs approximately 2.9 fg and contains six 16S rDNA copies.

To assess the relationship between the ‘absolute’ abundance of OTUs or genera with body odours, we transformed the relative abundance data, taking into account the bacterial load determined by qPCR (for details, see legend to Figure 7). This analysis greatly confirmed the observations based on the relative OTU abundance data and did not reveal additional taxa that correlated (Spearman *r* > 0.5 or < –0.5) with odours. Figure 7, for example, shows the correlation between the acid-spicy odour intensity and the ‘absolute’ abundance of *S. hominis* OTU154509. The ‘absolute’ abundance of this OTU above the median value was associated with an acid-spicy odour intensity >0.5 in all (22/22) instances, whereas an ‘absolute’ abundance below the median corresponded to an odour intensity <0.5 in 77% (17/22) of cases. In parallel, we performed pairwise comparisons of taxonomic profiles of individuals who differed in body odour intensity (high vs. low) but were matched in other parameters such as sex (females), the use of AP (non-AP users) and, as much as possible, physico-chemical characteristics (Additional file 14: Table S9). These pairwise comparisons (F2 vs. F26, F2 vs. F1 and F5 vs. F26) confirmed previous findings that individuals with higher odour intensities had a greater proportion of the genus *Corynebacterium*, mainly represented by OTU470219 assigned to *C. tuberculostearicum*, as well as a greater proportion of two *S. hominis* OTUs. In contrast, individuals with lower odours had a higher proportion of *P. acnes* OTU368907.

## Conclusions

To assess the role of bacterial communities in the formation of body odours, we studied axillary skin microbiota by using a culture-independent approach and correlated these data to olfactory analysis.

Not surprisingly, global sweat odour and bacterial load on armpit skin was higher in non-AP users in comparison to AP users. For non-AP users, global sweat odour intensity and fatty and acid-spicy odour descriptors were higher in males than in females. No direct correlations were observed between odour precursor concentration in underarms and sweat odours, which underlines the importance of bacteria in odour formation.

Co-occurrence analysis revealed putative mutualistic and competitive relationships at the genus and subgenus levels. Such interactions apparently affect the formation of body odours, which opens the possibility of developing alternative solutions to reduce body odours. For instance, an increase in bacteria that compete with odour-generating bacteria may provide a means to reduce body odours without significantly affecting overall microbiota abundance, bypassing the need to use APs or bactericidal substances. Here, we identified some candidates with deodorant potential among the bacteria that were negatively correlated with odours. However, these evaluations are only preliminary and should be confirmed with a larger cohort.

Culture-based methods have provided the basis to study the correlation between bacterial communities and body odours since the 1950s [40]. Research in this field is now progressing towards high-throughput culture-independent analyses of bacterial communities, allowing the determination not only of taxonomic abundances but also of the functional potential using metagenomics, metatranscriptomics [25] and metaproteomics.

## Methods

### Subject population

Twenty-six Caucasian subjects (13 females and 13 males) aged between 25 and 50 years old, employed and living in the Geneva area, were recruited. Two males were excluded from the analysis, as they were not present at all four sessions (morning and afternoon sessions for 2 days). Twenty-four subjects completed the study, consisting of 13 AP users (seven females and six males) and 11 non-AP users (six females and five males).

### Clinical study design

The clinical study was performed at Firmenich SA, Geneva, Switzerland, on October 20–22, 2010. The protocol was approved by the Firmenich Ethical Committee of Geneva. Experiments were undertaken with the understanding and written consent of each subject and in accordance with ethical principles, including those of the World Medical Association Declaration of Helsinki. Subjects signed a consent form (defined by Firmenich SA) to agree to the experimental protocol before the first day of bacterial sampling. Antibiotic treatments or (self-reported) skin problems during the clinical study were exclusion criteria. Only females with regular menstrual cycles were included. Participants completed a questionnaire about their medical treatments, hormonal contraceptive use, intake of vitamins and probiotics, smoking habits, menstrual history and axillary hair removal.

Subjects were asked to wear a 100% cotton T-shirt during experimental days, a new one every day. The following activities were restricted during the experiment: bathing, swimming, shaving axillae (restrictions started 16 h before the experiment), the use of protein food supplements or steroids and the use of body cosmetics and perfume other than the one supplied. Subjects were asked to use a neutral shower gel (supplied by Firmenich) 7 days before the beginning of the project and on each morning during the experiment. Morning showers were required during the 3-day experiment, whereas evening showers were optional. The subjects were asked to participate in the morning sessions not less than 1 h and not more than 2.5 h after the morning shower. They came during the first 2 days at fixed hours in the morning (sessions 1 and 3, from 8 to 10 a.m.) and the afternoon (sessions 2 and 4, 2 to 4 p.m.) with a 6-h interval between the two sessions. A code number was assigned to each subject. At each session, olfactory evaluation of the left and right axillae was carried out in a blinded fashion. The skin pH of the left axilla was recorded, and the right axillae were sampled for 16S rDNA analysis. Underarm sweat (left and right axillae) was collected on day 3 of the study for odour precursor quantification.

### pH measurement

The pH of the underarm skin surface was evaluated on the left axilla after the olfactory assessment. The equipment used was a device that combines the Skin-pH-meter 900 and the Sebumeter SM 810 (Courage-Khazaka, ELECTRONIC GmbH, Köln, Germany). The electrodes were carefully decontaminated with 70% ethanol and a distilled water solution between subjects.

### Olfactory assessment

The underarm odour assessments (left and right axillae) were carried out by four trained assessors (two women and two men). The blinded assessors were in individual cabins, whereas the subjects stood up outside the cabins. Assessors participated in eight training sessions, where they were asked to describe the odour of various chemicals and to evaluate their intensity in incubated human sweat and human axillae. Those chemicals corresponding to the nine descriptors used in our test were as follows: (0.05; 0.01; 0.002%) ((+−)-3-mercapto-3-methyl-1-hexanol): sulfury-cat urine-grapefruit-blackcurrant; (0.04; 0.2; 1%) (E/Z)-3-methyl-2-hexenoic acid and (+−)-3-hydroxy-3-methylhexanoic acid (1:1): acid-spicy-cumin; (0.4; 2; 10%) hexanoic acid, heptanoic acid, octanoic acid, nonanoic acid, decanoic acid (2:1:2:2:1): fatty-foot-wet/mould sponge; (0.02; 0.1; 0.5%) 4-ethyl octanoic acid: animalic-costus-goaty; (0.002; 0.01; 0.05%) 5α-androst-16-en-3-one: urinal-stale urine; (0.002; 0.01; 0.5%) butyric acid and isovaleric acids (1:1): vomit-rancid-cheese curd; (0.004; 0.02; 0.1%) 3-hydroxy-3-methyl-2,4-nonanedione: buttery-dairy; (0.002; 0.01; 0.005%) (+−)-3-mercapto-2-methyl-1-pentanol: fresh onion; (0.004; 0.02; 0.1%) p-cresol (para**-**hydroxytoluene): faecal-stable-barn. The training was performed by using (100 mL) smelling jars where 20 μL of the solutions was placed on smelling strips. The headspace was equilibrated overnight, and the jars were open briefly for the sniffing.

The global sweat odour intensity (sweat perception) along with the nine odour descriptors was evaluated by the four trained assessors following a linear scale from 0 (not perceptible) to 10 (very intense) on both underarms. Odour intensities were averaged for the four assessors.

### Sweat collection and analysis

**Scheme 1:**
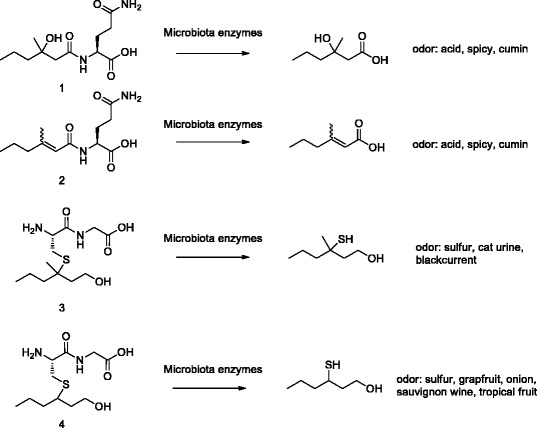
**Chemical structures of the most important odourless water soluble precursors secreted by apocrine glands and odorant compounds obtained by microbial activity.** Precursor **1**, *N*-α-3-hydroxy-3-methylhexanoyl-(L)-glutamine; precursor **2**, *N*-α-3-methylhexenoyl-(L)-glutamine; precursor **3**, *S*-[1-(2-hydroxyethyl)-1-methylbutyl]-(L)-cysteinylglycine; precursor **4**, *S*-[1-(2-hydroxyethyl)-butyl]-(L)-cysteinylglycine.

Subjects collected perspiration from their left and right axillae by staying in a sauna room facility for 10 min at midday on day 3 of the study. Sweat was collected by scrubbing the underarms with a 50-mL sterile Falcon tubes (BD Biosciences) and stored at 4°C for up to 1 h. Four hundred microlitres (350 μL for subject F6) of a sweat sample was acidified with 5 μL of trifluoroacetic acid and stored at −20°C until the analysis of odour precursors **1**–**4** (Scheme 1). Ultra-performance liquid chromatography coupled to a mass spectrometry was performed as described previously [22,27].

### Bacterial DNA sampling

The right axillae were swabbed with nylon-tipped swabs (Nylon Flocked Swab in a dry tube, Copan) moistened with a 150-μL solution of Tris-EDTA (TE) buffer (10 mM Tris, 1 mM EDTA, pH 8) and 0.5% (v/v) Tween-20. The skin surface was swabbed in two perpendicular directions within a sterile steel ring of 2.5 cm diameter. The swab head was cut from the stick with sterile scissors and placed in the inner tube of the Swab Extraction Tube System (SETS, Roche). Samples were centrifuged for 1 min at 6,000 g. The eluate (about 100 μL) was stored at −20°C. At each of the four sampling times (morning and afternoon sessions over 2 days), a negative control was performed by moistening a swab tip with TE (but without swabbing any surface).

### DNA extraction

DNA was extracted by using the NucleoSpin Soil KIT (Macherey-Nagel). The swab eluate, 700 μL of lysis buffer SL1 and 120 μL of Enhancer SX, was shaken in a NucleoSpin Bead Tube for 2 min at maximum speed on a Vortex-Genie 2 with a horizontal tube holder (Scientific Industries). From this point, we followed the protocol as described in the NucleoSpin Soil KIT booklet (January 2010/Rev. 01). DNA was eluted in 50 μL of elution buffer SE.

### PCR and pyrosequencing

The V1–3 region of the bacterial 16S rRNA gene, corresponding to *Escherichia coli* 16S rDNA positions 28–514 (excluding primer sequences), was amplified. The PCRs included 9 μL of DNA extract, 12 pmol of each forward primer 5′-ctatgcgccttgccagcccgctcag*ac*GAGTTTGATCMTGGCTCAG and a barcoded reverse primer 5′-cgtatcgcctccctcgcgccatcagNNNNNNNN*at*CCGCGGCTGCTGGCAC in 25 μL Primestar HS Premix (Takara). The composite PCR primers included the following: (i) the sequence of the broad range 16S rRNA gene forward or reverse primer (uppercase); (ii) a dinucleotide linker (lowercase, italic) introduced to prevent pairing of non-16S portions of the primers; (iii) a sample-specific eight-base sequence (NNNNNNNN) (Additional file 15: Table S10); and (iv) the 25-base sequence of the 454 Life Science Titanium Fusion Primer A or B (lowercase). For each sample, PCRs were carried out in duplicate for 40 cycles by using the following parameters: 98°C for 10 s, 60°C for 15 s and 72°C for 1 min. Two replicate PCRs were then pooled. The DNA concentration of amplicons of interest was determined on the 2100 Bioanalyzer (Agilent Technologies) by using a DNA 1000 lab chip. Fifty-nanogram DNA amplicons of each of the skin samples (<50 ng for the control samples) were pooled, and the amplicons of appropriate size were purified from the agarose gel by using the GFX PCR DNA and Gel Purification Kit (GE Healthcare). The 454 GS FLX Titanium Pyrosequencing from the Adaptor A (reverse primer) side was performed at the Functional Genomics Center Zurich (Zurich, Switzerland).

### qPCR

Bacterial loads in the skin swab samples were assessed by using qPCR targeting the 16S rRNA gene with universal primers. Quantitative PCR was run on an Mx3005P Stratagene cycler by using the Brilliant II SYBR Green QPCR Master Mix Kit (Stratagene). Reaction mixtures contained 1 μL of DNA extract, 7.5 pmol of each forward (5′-ACTCCTACGGGAGGCAGCAGT) and reverse (5′-ATTACCGCGGCTGCTGGC) HPLC-purified primers [41] and 0.75 μL of 1/250 diluted reference dye, in a total volume of 25 μL. These primers amplify the V3 region of the 16S rRNA gene (*E. coli* positions 338–534). The cycling conditions included initial denaturation of 10 min at 95°C followed by 40 cycles of 95°C for 30 s and 68° for 1 min. The reference curve for DNA quantitation was created by using a known concentration of genomic DNA of *S. aureus* strain MW2.

### Sequence analysis

Sequence filtering and analysis of sequence data was essentially performed with the MOTHUR (v. 1.30) software package [42]. We removed sequences (command trim.seqs) if they contained any of the following: (i) a mismatch in the barcode, (ii) more than one mismatch in the 16S sequence of the reverse primer, (iii) ambiguous bases or (iv) runs of ≥10 identical nucleotides. Sequences were truncated at the end of the last 50-nt window with an average Phred quality ≥35. Sequences that were <250 (after quality trimming) or >600 nt in length were discarded. The sequences that passed the steps (i–ii) were deposited in MG-RAST as a fastq file under the project ID 6526. Taxonomic assignments of phylotypes were made by OTU picking with the Greengenes reference database [43] pre-clustered at 97% using BLASTN and pipeline 6 as described previously [44]. The matching full-length reference 16S rRNA gene sequences and their taxonomic information were used in downstream analyses. OTUs identified in negative controls with a mean relative abundance higher than that found in the skin samples were subtracted from the sample data set prior to further analysis. Sequences belonging to several OTUs assigned to the genus *Staphylococcus* were further investigated using naïve Bayesian method [45] and the reference Greengenes taxonomy database (pre-clustered at 99% identity), with a confidence score threshold of 80% (command classify.seqs in MOTHUR).

### Clustering of bacterial communities

Microbiota from different samples were clustered by using principal coordinate analysis in PRIMER-E (Plymouth, UK) on the basis of (i) the Bray-Curtis similarity [46] of square-root-transformed OTU abundance and (ii) UniFrac distances [47]. The pre-constructed Greengenes reference OTU tree was used as the input file for the Fast UniFrac web interface [48].

### Statistical analysis

To determine significant differences between groups of samples, we used PERMANOVA [49] (in PRIMER-E package). To assess differences in the abundance of a particular taxon, odour descriptor, odour precursors, skin pH or skin bacterial load, we calculated *P* values on the basis of the Mann-Whitney *U* test. Spearman correlation coefficients were calculated by using the PRIMER-E package in order to estimate the significance of correlation between taxa, body odours and odour precursor concentration. Statistical significance was set at a 95% confidence level (*P* < 0.05). No corrections for multiple comparisons were performed.

## Additional files

Additional file 1: Table S1. Subjects’ characteristics, olfactory assessment and chemical analysis. Additional file 2: Figure S1. Comparisons of the left underarm pH at the four sessions. Thick horizontal lines represent the median values. T1: Morning session on day 1; T2: Afternoon session on day 1; T3: Morning session on day 2; T4: Afternoon session on day 2; F: Female; M: Male; AP: Antiperspirant user; Non-AP: Non-antiperspirant user. Additional file 3: Table S2. Statistically significant differences in the skin pH, bacterial loads and body odours between antiperspirant user types or sexes. Additional file 4: Figure S2. Chemical analysis of odour precursors in freshly collected odourless human sweat samples. The ratio (precursors 1 + 2)/(precursors 3 + 4) is given. Thick horizontal lines represent the median values. Sweat was collected from both the left and right axillae after the subject spent 10 min in a sauna on day 3. Mann-Whitney *U* test, *P* = 0.035 for all males (M) vs. all females (F). Non-AP: Non-antiperspirant user; AP: Antiperspirant user. Additional file 5: Table S3. Means and ranges of the intensities of the odour descriptors across the four assessors. Additional file 6: Figure S3. Comparison of global sweat intensity in the left and right underarms. Mean values obtained from the four trained assessors are presented for each individual and session. Odour intensity was evaluated on a scale from 0 to 10. Additional file 7: Table S4. Statistically significant differences in body odour intensity between morning and afternoon sessions. Additional file 8: Table S5. Assessment of the inhibitory effect on qPCR of DNA extracts from antiperspirant users. Additional file 9: Table S6. Operational taxonomic units (OTUs) identified in the control samples. Additional file 10: Figure S4. Percentage of putative contaminant 16S sequences as a function of bacterial DNA concentration in DNA extracts. DNA quantity in purified extracts was calculated by using *S. aureus* MW2 genomic DNA as a reference. The *S. aureus* MW2 genome weighs approximately 2.9 fg and contains six 16S rDNA copies. The analysed subjects were non-antiperspirant users. A trend line was added for clarity. Additional file 11: Table S7. Operational taxonomic unit (OTU) counts and relative abundance in normalized data set. Additional file 12: Figure S5. Ecological indices based on the relative abundance of operational taxonomic units (OTUs) in the normalized data set. (A) Number of OTUs identified. (B) Shannon diversity index [H’(loge)]. Thick horizontal lines represent the median values. T1: Morning session on day 1; T2: Afternoon session on day 1; T3: Morning session on day 2; T4: Afternoon session on day 2; F: Female; M: Male; Non-AP, Non-antiperspirant user. Additional file 13: Table S8. Co-occurrence and exclusion relationships between operational taxonomic units (OTUs), genera or phyla. Additional file 14: Table S9. Comparison of taxa abundance between selected female non-antiperspirant users. Additional file 15: Table S10. Sequences of eight-base barcodes for 454 pyrosequencing.

## Abbreviations

AP: Antiperspirant
OTU: Operational taxonomic unit

## References

[1] James AG, Austin CJ, Cox DS, Taylor D, Calvert R (2013). Microbiological and biochemical origins of human axillary odour. FEMS Microbiol Ecol.

[2] Leyden JJ, McGinley KJ, Holzle E, Labows JN, Kligman AM (1981). The microbiology of the human axilla and its relationship to axillary odor. J Invest Dermatol.

[3] Taylor D, Daulby A, Grimshaw S, James G, Mercer J, Vaziri S (2003). Characterization of the microflora of the human axilla. Int J Cosmet Sci.

[4] Shelley WB, Hurley HJ (1953). The physiology of the human axillary apocrine sweat gland. J Invest Dermatol.

[5] Choudhry R, Hodgins MB, Van der Kwast TH, Brinkmann AO, Boersma WJ (1992). Localization of androgen receptors in human skin by immunohistochemistry: implications for the hormonal regulation of hair growth, sebaceous glands and sweat glands. J Endocrinol.

[6] Stoddart DM (1990). The Scented Ape: the Biology and Culture of Human Odour.

[7] Jackman PJ, Noble WC (1983). Normal axillary skin microflora in various populations. Clin Exp Dermatol.

[8] Doty RL, Orndorff MM, Leyden J, Kligman A (1978). Communication of gender from human axillary odors: relationship to perceived intensity and hedonicity. Behav Biol.

[9] Brody B (1975). The sexual significance of the axillae. Psychiatry.

[10] Montagna W, Parakkal PF (1974). Apocrine glands. The Structure and Function of Skin.

[11] Kuhn F, Natsch A (2009). Body odour of monozygotic human twins: a common pattern of odorant carboxylic acids released by a bacterial aminoacylase from axilla secretions contributing to an inherited body odour type. J R Soc Interface.

[12] Roberts SC, Gosling LM, Spector TD, Miller P, Penn DJ, Petrie M (2005). Body odor similarity in noncohabiting twins. Chem Senses.

[13] Wedekind C, Furi S (1997). Body odour preferences in men and women: do they aim for specific MHC combinations or simply heterozygosity?. Proc Biol Sci.

[14] Wedekind C, Seebeck T, Bettens F, Paepke AJ (1995). MHC-dependent mate preferences in humans. Proc Biol Sci.

[15] Toyoda Y, Sakurai A, Mitani Y, Nakashima M, Yoshiura K, Nakagawa H (2009). Earwax, osmidrosis, and breast cancer: why does one SNP (538G > A) in the human ABC transporter ABCC11 gene determine earwax type?. FASEB J.

[16] Knaggs HE, Wood EJ, Rizer RL, Mills OH (2004). Post-adolescent acne. Int J Cosmet Sci.

[17] Holland KT, Bojar RA (2002). Cosmetics: what is their influence on the skin microflora?. Am J Clin Dermatol.

[18] Sulzberger MB, Zak FG, Herrmann F (1949). Studies of sweating; on the mechanism of action of local antiperspirants. Arch Derm Syphilol.

[19] Havlíček J, Dvořáková R, Bartoš L, Flegr J (2006). Non-advertised does not mean concealed: body odour changes across the human menstrual cycle. Ethology.

[20] Natsch A, Gfeller H, Gygax P, Schmid J, Acuna G (2003). A specific bacterial aminoacylase cleaves odorant precursors secreted in the human axilla. J Biol Chem.

[21] Natsch A, Schmid J, Flachsmann F (2004). Identification of odoriferous sulfanylalkanols in human axilla secretions and their formation through cleavage of cysteine precursors by a C-S lyase isolated from axilla bacteria. Chem Biodivers.

[22] Starkenmann C, Niclass Y, Troccaz M, Clark AJ (2005). Identification of the precursor of (S)-3-methyl-3-sulfanylhexan-1-ol, the sulfury malodour of human axilla sweat. Chem Biodivers.

[23] Emter R, Natsch A (2008). The sequential action of a dipeptidase and a beta-lyase is required for the release of the human body odorant 3-methyl-3-sulfanylhexan-1-ol from a secreted Cys-Gly-(S) conjugate by Corynebacteria. J Biol Chem.

[24] Schmid-Wendtner M-H, Korting HC (2007). pH and Skin Care.

[25] Fredrich E, Barzantny H, Brune I, Tauch A (2013). Daily battle against body odor: towards the activity of the axillary microbiota. Trends Microbiol.

[26] Starkenmann C, Mayenzet F, Brauchli R, Troccaz M (2013). 5alpha-Androst-16-en-3alpha-ol beta-D-glucuronide, precursor of 5alpha-androst-16-en-3alpha-ol in human sweat. Chem Biodivers.

[27] Troccaz M, Borchard G, Vuilleumier C, Raviot-Derrien S, Niclass Y, Beccucci S (2009). Gender-specific differences between the concentrations of nonvolatile (R)/(S)-3-methyl-3-sulfanylhexan-1-ol and (R)/(S)-3-hydroxy-3-methyl-hexanoic acid odor precursors in axillary secretions. Chem Senses.

[28] Biesbroek G, Sanders EA, Roeselers G, Wang X, Caspers MP, Trzcinski K (2012). Deep sequencing analyses of low density microbial communities: working at the boundary of accurate microbiota detection. PLoS ONE.

[29] Willner D, Daly J, Whiley D, Grimwood K, Wainwright CE, Hugenholtz P (2012). Comparison of DNA extraction methods for microbial community profiling with an application to pediatric bronchoalveolar lavage samples. PLoS ONE.

[30] Grice EA, Kong HH, Conlan S, Deming CB, Davis J, Young AC (2009). Topographical and temporal diversity of the human skin microbiome. Science.

[31] Zeeuwen PL, Boekhorst J, van den Bogaard EH, de Koning HD, van de Kerkhof PM, Saulnier DM (2012). Microbiome dynamics of human epidermis following skin barrier disruption. Genome Biol.

[32] Costello EK, Lauber CL, Hamady M, Fierer N, Gordon JI, Knight R (2009). Bacterial community variation in human body habitats across space and time. Science.

[33] Egert M, Schmidt I, Hohne HM, Lachnit T, Schmitz RA, Breves R (2011). rRNA-based profiling of bacteria in the axilla of healthy males suggests right-left asymmetry in bacterial activity. FEMS Microbiol Ecol.

[34] Grice EA, Kong HH, Renaud G, Young AC, Bouffard GG, Blakesley RW (2008). A diversity profile of the human skin microbiota. Genome Res.

[35] Fierer N, Hamady M, Lauber CL, Knight R (2008). The influence of sex, handedness, and washing on the diversity of hand surface bacteria. Proc Natl Acad Sci U S A.

[36] Lozupone C, Lladser ME, Knights D, Stombaugh J, Knight R (2011). UniFrac: an effective distance metric for microbial community comparison. ISME J.

[37] Staudinger T, Pipal A, Redl B (2011). Molecular analysis of the prevalent microbiota of human male and female forehead skin compared to forearm skin and the influence of make-up. J Appl Microbiol.

[38] Tauch A, Kaiser O, Hain T, Goesmann A, Weisshaar B, Albersmeier A (2005). Complete genome sequence and analysis of the multiresistant nosocomial pathogen *Corynebacterium jeikeium* K411, a lipid-requiring bacterium of the human skin flora. J Bacteriol.

[39] Barzantny H, Brune I, Tauch A (2012). Molecular basis of human body odour formation: insights deduced from corynebacterial genome sequences. Int J Cosmet Sci.

[40] Shelley WB, Hurley HJ, Nichols AC (1953). Axillary odor; experimental study of the role of bacteria, apocrine sweat, and deodorants. AMA Arch Derm Syphilol.

[41] Hartman AL, Lough DM, Barupal DK, Fiehn O, Fishbein T, Zasloff M (2009). Human gut microbiome adopts an alternative state following small bowel transplantation. Proc Natl Acad Sci U S A.

[42] Schloss PD, Westcott SL, Ryabin T, Hall JR, Hartmann M, Hollister EB (2009). Introducing mothur: open-source, platform-independent, community-supported software for describing and comparing microbial communities. Appl Environ Microbiol.

[43] McDonald D, Price MN, Goodrich J, Nawrocki EP, DeSantis TZ, Probst A (2012). An improved Greengenes taxonomy with explicit ranks for ecological and evolutionary analyses of bacteria and archaea. ISME J.

[44] Lazarevic V, Gaia N, Girard M, Francois P, Schrenzel J (2013). Comparison of DNA extraction methods in analysis of salivary bacterial communities. PLoS ONE.

[45] Wang Q, Garrity GM, Tiedje JM, Cole JR (2007). Naive Bayesian classifier for rapid assignment of rRNA sequences into the new bacterial taxonomy. Appl Environ Microbiol.

[46] Bray R, Curtis JT (1957). An ordination of the upland forest communities of southern Wisconsin. Ecol Monograph.

[47] Lozupone C, Knight R (2005). UniFrac: a new phylogenetic method for comparing microbial communities. Appl Environ Microbiol.

[48] Hamady M, Lozupone C, Knight R (2010). Fast UniFrac: facilitating high-throughput phylogenetic analyses of microbial communities including analysis of pyrosequencing and PhyloChip data. ISME J.

[49] Clarke KR (1993). Non-parametric multivariate analyses of changes in community structure. Austr J Ecol.

