# Supplementary figures and images for: Mapping axillary microbiota responsible for body odours using a culture-independent approach
Source: Microbiome. 2015 Jan 24;3:3. doi: 10.1186/s40168-014-0064-3 (PMC4316401; doi:10.1186/s40168-014-0064-3)

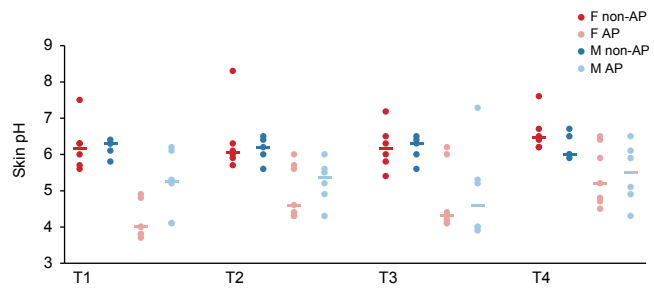

Supplement: Additional file 2: Figure S1. — Comparisons of the left underarm pH at the four sessions. Thick horizontal lines represent the median values. T1: Morning session on day 1; T2: Afternoon session on day 1; T3: Morning session on day 2; T4: Afternoon session on day 2; F: Female; M: Male; AP: Antiperspirant user; Non-AP: Non-antiperspirant user. [file 40168_2014_64_MOESM2_ESM.pdf]

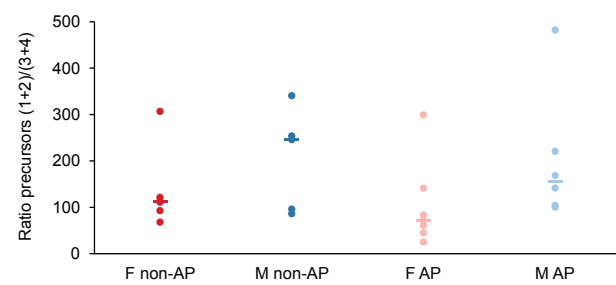

Supplement: Additional file 4: Figure S2. — Chemical analysis of odour precursors in freshly collected odourless human sweat samples. The ratio (precursors 1 + 2)/(precursors 3 + 4) is given. Thick horizontal lines represent the median values. Sweat was collected from both the left and right axillae after the subject spent 10 min in a sauna on day 3. Mann-Whitney U test, P = 0.035 for all males (M) vs. all females (F). Non-AP: Non-antiperspirant user; AP: Antiperspirant user. [file 40168_2014_64_MOESM4_ESM.pdf]

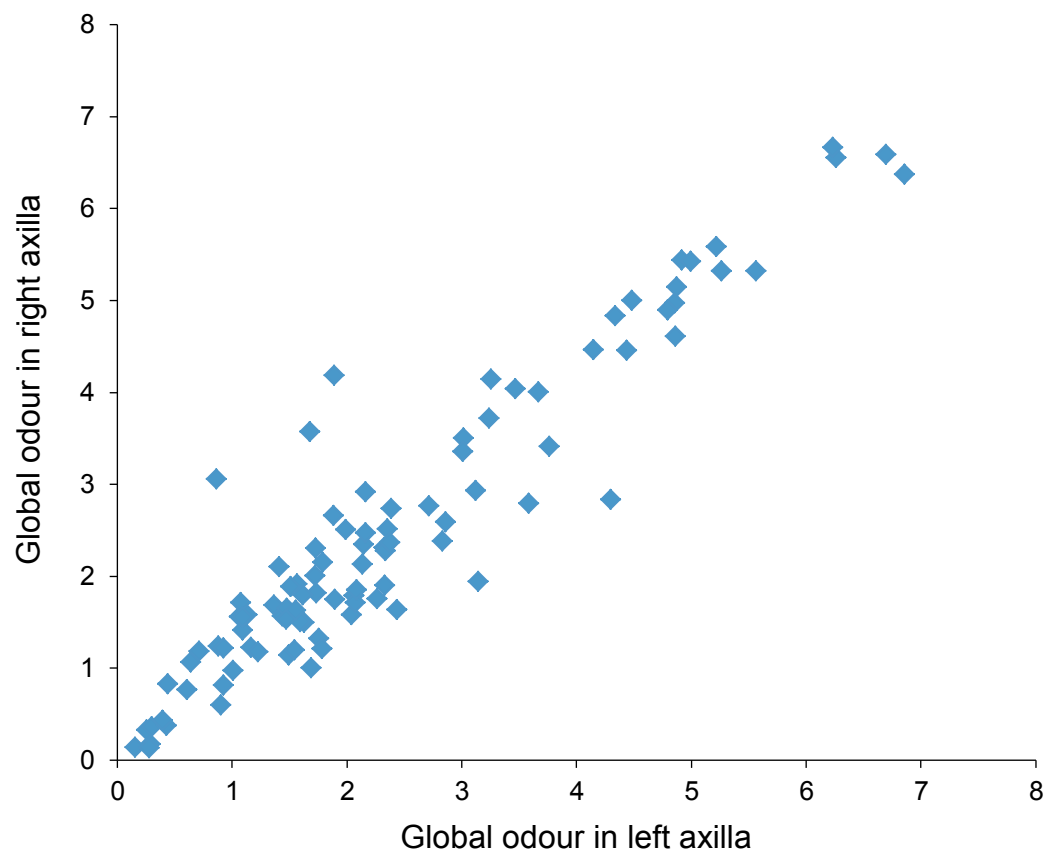

Supplement: Additional file 6: Figure S3. — Comparison of global sweat intensity in the left and right underarms. Mean values obtained from the four trained assessors are presented for each individual and session. Odour intensity was evaluated on a scale from 0 to 10. [file 40168_2014_64_MOESM6_ESM.pdf]

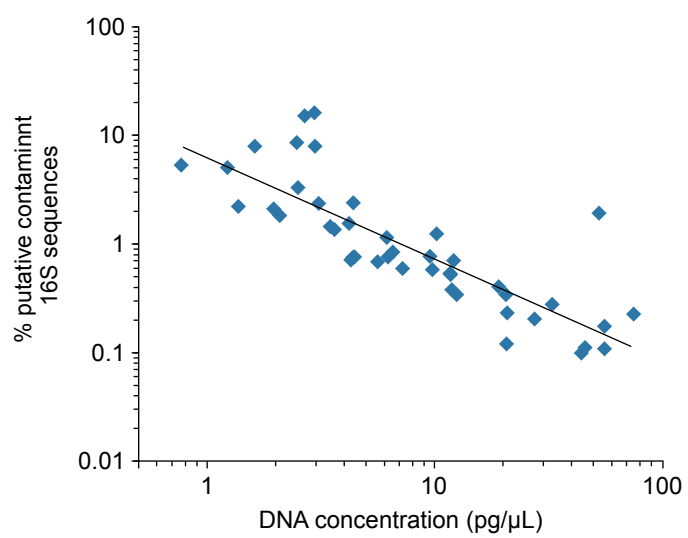

Supplement: Additional file 10: Figure S4. — Percentage of putative contaminant 16S sequences as a function of bacterial DNA concentration in DNA extracts. DNA quantity in purified extracts was calculated by using S. aureus MW2 genomic DNA as a reference. The S. aureus MW2 genome weighs approximately 2.9 fg and contains six 16S rDNA copies. The analysed subjects were non-antiperspirant users. A trend line was added for clarity. [file 40168_2014_64_MOESM10_ESM.pdf]

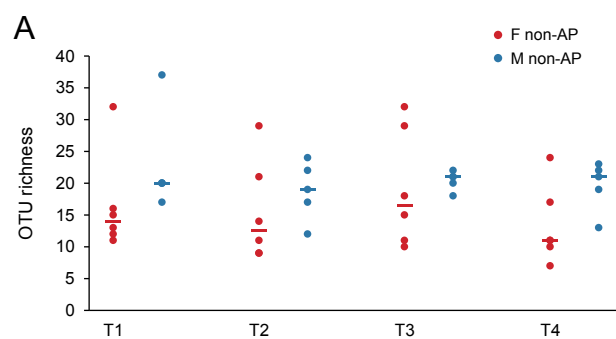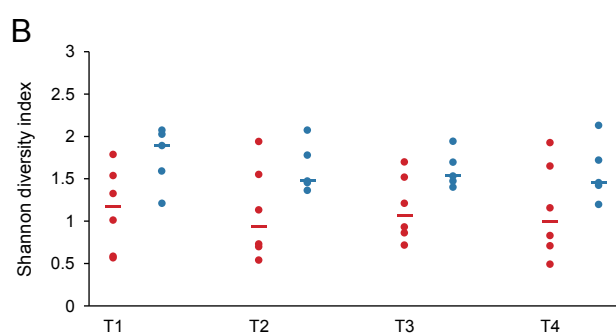

Supplement: Additional file 12: Figure S5. — Ecological indices based on the relative abundance of operational taxonomic units (OTUs) in the normalized data set. (A) Number of OTUs identified. (B) Shannon diversity index [H’(loge)]. Thick horizontal lines represent the median values. T1: Morning session on day 1; T2: Afternoon session on day 1; T3: Morning session on day 2; T4: Afternoon session on day 2; F: Female; M: Male; Non-AP, Non-antiperspirant user. [file 40168_2014_64_MOESM12_ESM.pdf]
