# Supplementary material for: Mapping axillary microbiota responsible for body odours using a culture-independent approach
Source: Microbiome. 2015 Jan 24;3:3. doi: 10.1186/s40168-014-0064-3 (PMC4316401; doi:10.1186/s40168-014-0064-3)
Supplement: Additional file 3: Table S2. — Statistically significant differences in the skin pH, bacterial loads and body odours between antiperspirant user types or sexes. [file 40168_2014_64_MOESM3_ESM.docx]

**Table S2. Statistically significant differences in the skin pH, bacterial loads and body odours between antiperspirant user types or sexes.**

| **Comparison** | **Session** | ***P* value^a^** | | | | | |
| --- | --- | --- | --- | --- | --- | --- | --- |
|  |  | **pH** | **Bacterial load** | **Odour** | | | |
|  |  |  |  | **Sulfury-cat urine** | **Fatty** | **Fresh onion** | **Global** |
| **F_AP vs  M_AP** | **T1** | **0.018** | 0.53 |  |  |  |  |
|  | **T2** | 0.72 | 1 |  |  |  |  |
|  | **T3** | 0.62 | 0.45 |  |  |  |  |
|  | **T4** | 0.83 | **0.035** |  |  |  |  |
| **(F+M)_AP vs (F+M)_non-AP** | **T1** | **0.00035** | **0.0000008** | **0.0048** |  | **0.0012** | **0.0015** |
|  | **T2** | **0.00043** | **0.0000032** | **0.015** |  | **0.019** | **0.015** |
|  | **T3** | **0.0031** | **0.0000008** | 0.052 |  | 0.49 | 0.12 |
|  | **T4** | **0.0096** | **0.0000008** | **0.018** |  | 0.22 | **0.047** |
| **F_AP vs  F_non-AP** | **T1** | **0.0033** | **0.0012** |  |  | **0.038** |  |
|  | **T2** | **0.012** | **0.0023** |  |  | 0.57 |  |
|  | **T3** | **0.032** | **0.0012** |  |  | 0.89 |  |
|  | **T4** | 0.073 | **0.0012** |  |  | 0.94 |  |
| **M_AP vs  M_non-AP** | **T1** | **0.043** | **0.0043** | **0.0087** |  | **0.034** | **0.0043** |
|  | **T2** | **0.022** | **0.0043** | **0.017** |  | **0.008** | **0.0043** |
|  | **T3** | 0.082 | **0.0043** | 0.052 |  | 0.18 | **0.030** |
|  | **T4** | 0.17 | **0.0043** | **0.03** |  | 0.17 | 0.052 |
| **F_(AP+non-AP) vs M_(AP+non-AP)** | **T1** |  |  |  | **0.015** |  |  |
|  | **T2** |  |  |  | 0.3 |  |  |
|  | **T3** |  |  |  | 0.23 |  |  |
|  | **T4** |  |  |  | 0.9 |  |  |
| **F_non-AP vs M_non-AP** | **T1** |  |  |  | **0.0087** |  |  |
|  | **T2** |  |  |  | 0.052 |  |  |
|  | **T3** |  |  |  | 0.052 |  |  |
|  | **T4** |  |  |  | 0.66 |  |  |

F, female; M, male; AP, antiperspirant user; non-AP, non-antiperspirant user; T1, morning session on day 1; T2, afternoon session on day 1; T3, morning session on day 2; T4, afternoon session on day 2.

**^a^** Statistical analysis used was the Mann-Whitney U test. P values are reported for all sampling points whenever the difference between the groups compared was statistically significant in at least one time point. Significant P values (<0.05) are given in bold.
