# Supplementary material for: Mapping axillary microbiota responsible for body odours using a culture-independent approach
Source: Microbiome. 2015 Jan 24;3:3. doi: 10.1186/s40168-014-0064-3 (PMC4316401; doi:10.1186/s40168-014-0064-3)
Supplement: Additional file 5: Table S3. — Means and ranges of the intensities of the odour descriptors across the four assessors. [file 40168_2014_64_MOESM5_ESM.docx]

**Table S3. Means and ranges of the intensities of the odour descriptors across the four assessors.**

| **Assessor ID** | **Odour intensity** | | | | | | | | | | | | | | |
| --- | --- | --- | --- | --- | --- | --- | --- | --- | --- | --- | --- | --- | --- | --- | --- |
|  | **Sulfury-cat urine** | | | **Fatty** | | | **Acid-spicy** | | | **Fresh onion** | | | **Global** | | |
|  | **Min** | **Max** | **Mean** | **Min** | **Max** | **Mean** | **Min** | **Max** | **Mean** | **Min** | **Max** | **Mean** | **Min** | **Max** | **Mean** |
| **Assessor_1** | 0.02 | 7.13 | 0.52 | 0.02 | 3.61 | 0.40 | 0.01 | 5.00 | 0.14 | 0.02 | 7.79 | 0.55 | 0.04 | 7.97 | 2.48 |
| **Assessor_2** | 0.01 | 5.76 | 0.87 | 0.02 | 6.79 | 1.50 | 0.02 | 6.91 | 1.26 | 0.00 | 6.29 | 0.22 | 0.04 | 6.73 | 2.24 |
| **Assessor_3** | 0.02 | 7.61 | 1.20 | 0.02 | 8.81 | 1.07 | 0.00 | 5.23 | 0.68 | 0.03 | 9.13 | 1.70 | 0.00 | 9.07 | 2.86 |
| **Assessor_4** | 0.00 | 7.66 | 1.22 | 0.00 | 3.54 | 0.49 | 0.00 | 5.34 | 0.90 | 0.00 | 3.83 | 0.15 | 0.03 | 9.27 | 2.23 |

Odour intensity was evaluated on a scale from 0 to 10. Minimum (Min) and maximum (Max) values obtained by each assessor are given.
