# Supplementary material for: Mapping axillary microbiota responsible for body odours using a culture-independent approach
Source: Microbiome. 2015 Jan 24;3:3. doi: 10.1186/s40168-014-0064-3 (PMC4316401; doi:10.1186/s40168-014-0064-3)
Supplement: Additional file 7: Table S4. — Statistically significant differences in body odour intensity between morning and afternoon sessions. [file 40168_2014_64_MOESM7_ESM.docx]

**Table S4. Statistically significant differences in body odour intensity between morning and afternoon sessions.**

| **Group of subjects** | **Comparison** | ***P* value^a^ for the change in odour intensity** | | | |
| --- | --- | --- | --- | --- | --- |
|  |  | **Sulfury-cat urine** | **Fresh onion** | **Acid-spicy** | **Global** |
| **(F+M)_AP** | **T2 vs T1** | 0.72 | 0.76 |  | 0.11 |
|  | **T4 vs T3** | **0.033** | **0.048** |  | **0.017** |
| **(F+M)_non-AP** | **T2 vs T1** |  |  |  | 0.87 |
|  | **T4 vs T3** |  |  |  | **0.047** |
| **(F+M)_(AP+non-AP)** | **T2 vs T1** | 0.81 | 0.73 | 0.11 | 0.36 |
|  | **T4 vs T3** | **0.0044** | **0.0032** | **0.0099** | **0.0014** |
| **F_AP** | **T2 vs T1** |  | 0.9 |  | **0.0038** |
|  | **T4 vs T3** |  | **0.035** |  | 0.08 |
| **F_(AP+non-AP)** | **T2 vs T1** | 0.29 | 0.41 |  | 0.13 |
|  | **T4 vs T3** | **0.026** | **0.0052** |  | **0.013** |

F, female; M, male; AP, antiperspirant user; non-AP, non-antiperspirant user; T1, morning session on day 1; T2, afternoon session on day 1; T3, morning session on day 2; T4, afternoon session on day 2.

**^a^** Statistical analysis used was the Mann-Whitney U test. *P* values are reported for days 1 and 2 whenever the difference between the morning and afternoon sessions was statistically significant in at least one of the two days. Significant *P* values (<0.05) are given in bold.
