# Supplementary material for: Mapping axillary microbiota responsible for body odours using a culture-independent approach
Source: Microbiome. 2015 Jan 24;3:3. doi: 10.1186/s40168-014-0064-3 (PMC4316401; doi:10.1186/s40168-014-0064-3)
Supplement: Additional file 8: Table S5. — Assessment of the inhibitory effect on qPCR of DNA extracts from antiperspirant users. [file 40168_2014_64_MOESM8_ESM.docx]

**Table S5. Assessment of the inhibitory effect on qPCR of DNA extracts from antiperspirant users.**

| **Sample(s) [Subject_Session_antiperspirant use]^a^** | **Cycle threshold (Ct)^b^** |
| --- | --- |
| M21_1_non-AP | 19.89 |
| M21_1_non-AP + F15_1_AP | 19.93 |
| F15_1_AP | 25.52 |
| M25_1_non-AP | 20.08 |
| M25_1_non-AP + F16_1_AP | 20.08 |
| F16_1_AP | 25.06 |

F, female; M, male; AP, antiperspirant user; non-AP, non-antiperspirant user; Session 1, morning session on day 1.

^a^ One microlitre of each sample was used in qPCR.

^b^ Average for duplicate samples. Relative deviations from the average were <0.1%.
