# Supplementary material for: Mapping axillary microbiota responsible for body odours using a culture-independent approach
Source: Microbiome. 2015 Jan 24;3:3. doi: 10.1186/s40168-014-0064-3 (PMC4316401; doi:10.1186/s40168-014-0064-3)
Supplement: Additional file 13: Table S8. — Co-occurrence and exclusion relationships between operational taxonomic units (OTUs), genera or phyla. [file 40168_2014_64_MOESM13_ESM.docx]

**Table S8. Co-occurrence and exclusion relationships between operational taxonomic units (OTUs), genera or phyla.**

| **Taxon I** | | | | **Taxon II** | | | | ***r*** |
| --- | --- | --- | --- | --- | --- | --- | --- | --- |
| **Phylum; Genus or species; OTU** | **Relative abundance** | | **Positive  samples** | **Phylum; Genus or species; OTU** | **Relative abundance** | | **Positive  samples** |  |
|  | **Mean** | **Median** |  |  | **Mean** | **Median** |  |  |
| Firmicutes; Staphylococcus hominis; OTU154509 | 21.51 | 13.47 | 42 | Firmicutes; Staphylococcus hominis; OTU173469 | 2.64 | 1.36 | 33 | 0.960 |
| Firmicutes; Staphylococcus hominis; OTU154509 | 21.51 | 13.47 | 42 | Firmicutes; Staphylococcus hominis; OTU338191 | 0.18 | 0.10 | 25 | 0.887 |
| Firmicutes; Staphylococcus hominis; OTU173469 | 2.64 | 1.36 | 33 | Firmicutes; Staphylococcus hominis; OTU338191 | 0.18 | 0.10 | 25 | 0.866 |
| Firmicutes; Staphylococcus; OTU356733* | 38.77 | 32.41 | 44 | Firmicutes; Staphylococcus; OTU330679* | 5.24 | 3.87 | 40 | 0.838 |
| Actinobacteria; Corynebacterium; OTU416589 | 0.96 | 0.00 | 13 | Actinobacteria; Corynebacterium; OTU494493 | 0.19 | 0.00 | 10 | 0.823 |
| Actinobacteria; Corynebacterium; OTU13430 | 0.44 | 0.00 | 13 | Actinobacteria; Corynebacterium; OTU494493 | 0.19 | 0.00 | 10 | 0.734 |
| Actinobacteria; Corynebacterium; OTU264597 | 1.63 | 0.20 | 26 | Actinobacteria; Corynebacterium; OTU423737 | 0.46 | 0.00 | 16 | 0.700 |
| Firmicutes; Finegoldia; OTU86757 | 0.29 | 0.00 | 17 | Firmicutes; Dialister; OTU128382 | 0.11 | 0.00 | 8 | 0.686 |
| Actinobacteria; Corynebacterium tuberculostearicum; OTU470219 | 8.74 | 5.83 | 41 | Actinobacteria; Corynebacterium; OTU264597 | 1.63 | 0.20 | 26 | 0.650 |
| Actinobacteria; Corynebacterium; OTU416589 | 0.96 | 0.00 | 13 | Actinobacteria; Corynebacterium; OTU13430 | 0.44 | 0.00 | 13 | 0.640 |
| Actinobacteria; Propionibacterium granulosum; OTU12724 | 0.45 | 0.00 | 18 | Firmicutes; Staphylococcus haemolyticus; OTU354779 | 0.35 | 0.00 | 9 | 0.632 |
| Actinobacteria; Corynebacterium; OTU264597 | 1.63 | 0.20 | 26 | Firmicutes; Anaerococcus; OTU173036 | 1.39 | 0.00 | 20 | 0.627 |
| Actinobacteria; Corynebacterium; OTU264597 | 1.63 | 0.20 | 26 | Actinobacteria; Corynebacterium; OTU494493 | 0.19 | 0.00 | 10 | 0.626 |
| Firmicutes; Finegoldia; OTU86757 | 0.29 | 0.00 | 17 | Actinobacteria; Actinomyces neuii; OTU131783 | 0.02 | 0.00 | 6 | 0.615 |
| Firmicutes; Staphylococcus; OTU269541* | 0.25 | 0.00 | 14 | Firmicutes; Staphylococcus; OTU107105 | 0.08 | 0.00 | 4 | 0.602 |
| Firmicutes; Staphylococcus; OTU269541* | 0.25 | 0.00 | 14 | Firmicutes; Staphylococcus; OTU244892 | 0.08 | 0.00 | 4 | 0.602 |
| Firmicutes; Anaerococcus; OTU173036 | 1.39 | 0.00 | 20 | Actinobacteria; Corynebacterium; OTU494493 | 0.19 | 0.00 | 10 | 0.597 |
| Firmicutes; Peptoniphilus asaccharolyticus; OTU489717 | 0.34 | 0.00 | 14 | Actinobacteria; Corynebacterium simulans; OTU381339 | 0.12 | 0.00 | 4 | 0.595 |
| Firmicutes; Peptoniphilus asaccharolyticus; OTU489717 | 0.34 | 0.00 | 14 | Bacteroidetes; Porphyromonas; OTU268540 | 0.15 | 0.00 | 4 | 0.593 |
| Firmicutes; Peptoniphilus asaccharolyticus; OTU489717 | 0.34 | 0.00 | 14 | Actinobacteria; Kocuria palustris; OTU567669 | 0.01 | 0.00 | 4 | 0.588 |
| Actinobacteria; Corynebacterium; OTU423737 | 0.46 | 0.00 | 16 | Firmicutes; Anaerococcus; OTU357533 | 0.06 | 0.00 | 5 | 0.579 |
| Actinobacteria; Corynebacterium; OTU264597 | 1.63 | 0.20 | 26 | Actinobacteria; Corynebacterium; OTU13430 | 0.44 | 0.00 | 13 | 0.576 |
| Actinobacteria; Corynebacterium; OTU13430 | 0.44 | 0.00 | 13 | Actinobacteria; Corynebacterium lipophiloflavum; OTU470207 | 0.01 | 0.00 | 4 | 0.567 |
| Actinobacteria; Corynebacterium tuberculostearicum; OTU470219 | 8.74 | 5.83 | 41 | Actinobacteria; Corynebacterium; OTU494493 | 0.19 | 0.00 | 10 | 0.565 |
| Actinobacteria; Propionibacterium acnes; OTU107891 | 0.52 | 0.20 | 27 | Firmicutes; Peptoniphilus asaccharolyticus; OTU489717 | 0.34 | 0.00 | 14 | 0.562 |
| Actinobacteria; Corynebacterium; OTU494493 | 0.19 | 0.00 | 10 | Firmicutes; Staphylococcus cohnii; OTU326116 | 0.02 | 0.00 | 5 | 0.559 |
| Actinobacteria; Propionibacterium granulosum; OTU12724 | 0.45 | 0.00 | 18 | Firmicutes; Staphylococcus; OTU217912* | 0.03 | 0.00 | 6 | 0.558 |
| Actinobacteria; Propionibacterium granulosum; OTU12724 | 0.45 | 0.00 | 18 | Firmicutes; Staphylococcus; OTU269541* | 0.25 | 0.00 | 14 | 0.555 |
| Firmicutes; Staphylococcus; OTU330679* | 5.24 | 3.87 | 40 | Actinobacteria; Propionibacterium; OTU107891 | 0.52 | 0.20 | 27 | 0.554 |
| Actinobacteria; Corynebacterium; OTU264597 | 1.63 | 0.20 | 26 | Firmicutes; Staphylococcus hominis; OTU338191 | 0.18 | 0.10 | 25 | 0.553 |
| Actinobacteria; Corynebacterium; OTU423737 | 0.46 | 0.00 | 16 | Actinobacteria; Corynebacterium; OTU138110 | 0.09 | 0.00 | 4 | 0.552 |
| Actinobacteria; Corynebacterium; OTU423737 | 0.46 | 0.00 | 16 | Actinobacteria; Corynebacterium; OTU545862 | 0.19 | 0.00 | 4 | 0.551 |
| Actinobacteria; Corynebacterium; OTU13430 | 0.44 | 0.00 | 13 | Firmicutes; Staphylococcus cohnii; OTU326116 | 0.02 | 0.00 | 5 | 0.542 |
| Actinobacteria; Corynebacterium; OTU494493 | 0.19 | 0.00 | 10 | Actinobacteria; Corynebacterium lipophiloflavum; OTU470207 | 0.01 | 0.00 | 4 | 0.541 |
| Actinobacteria; Corynebacterium; OTU264597 | 1.63 | 0.20 | 26 | Actinobacteria; Corynebacterium; OTU416589 | 0.96 | 0.00 | 13 | 0.534 |
| Firmicutes; Staphylococcus hominis; OTU173469 | 2.64 | 1.36 | 33 | Actinobacteria; Corynebacterium; OTU423737 | 0.46 | 0.00 | 16 | 0.531 |
| Actinobacteria; Corynebacterium tuberculostearicum; OTU470219 | 8.74 | 5.83 | 41 | Actinobacteria; Corynebacterium; OTU13430 | 0.44 | 0.00 | 13 | 0.531 |
| Actinobacteria; Propionibacterium acnes; OTU368907 | 12.49 | 0.55 | 35 | Firmicutes; Staphylococcus; OTU269541* | 0.25 | 0.00 | 14 | 0.528 |
| Firmicutes; Anaerococcus; OTU173036 | 1.39 | 0.00 | 20 | Actinobacteria; Corynebacterium; OTU416589 | 0.96 | 0.00 | 13 | 0.527 |
| Actinobacteria; Corynebacterium; OTU416589 | 0.96 | 0.00 | 13 | Actinobacteria; Corynebacterium lipophiloflavum; OTU470207 | 0.01 | 0.00 | 4 | 0.525 |
| Firmicutes; Anaerococcus; OTU14290 | 0.54 | 0.10 | 27 | Firmicutes; Peptoniphilus asaccharolyticus; OTU489717 | 0.34 | 0.00 | 14 | 0.525 |
| Firmicutes; Anaerococcus; OTU173036 | 1.39 | 0.00 | 20 | Actinobacteria; Corynebacterium; OTU13430 | 0.44 | 0.00 | 13 | 0.522 |
| Actinobacteria; Corynebacterium; OTU423737 | 0.46 | 0.00 | 16 | Firmicutes; Staphylococcus hominis; OTU338191 | 0.18 | 0.10 | 25 | 0.520 |
| Firmicutes; Peptoniphilus asaccharolyticus; OTU489717 | 0.34 | 0.00 | 14 | Proteobacteria; Bacteroides ureolyticus; OTU386273 | 0.05 | 0.00 | 6 | 0.516 |
| Firmicutes; Staphylococcus hominis; OTU154509 | 21.51 | 13.47 | 42 | Actinobacteria; Corynebacterium; OTU423737 | 0.46 | 0.00 | 16 | 0.513 |
| Firmicutes; Staphylococcus hominis; OTU173469 | 2.64 | 1.36 | 33 | Actinobacteria; Corynebacterium; OTU264597 | 1.63 | 0.20 | 26 | 0.510 |
| Actinobacteria; Corynebacterium; OTU416589 | 0.96 | 0.00 | 13 | Firmicutes; Anaerococcus; OTU14290 | 0.54 | 0.10 | 27 | 0.508 |
| Firmicutes; Finegoldia; OTU86757 | 0.29 | 0.00 | 17 | Proteobacteria; Amaricoccus; OTU5804 | 0.09 | 0.00 | 4 | 0.505 |
| Firmicutes; Staphylococcus hominis; OTU154509 | 21.51 | 13.47 | 42 | Actinobacteria; Corynebacterium; OTU264597 | 1.63 | 0.20 | 26 | 0.504 |
| Firmicutes; Staphylococcus; OTU269541* | 0.25 | 0.00 | 14 | Firmicutes; Staphylococcus; OTU519827 | 0.01 | 0.00 | 3 | 0.503 |
| Firmicutes; Staphylococcus; OTU269541* | 0.25 | 0.00 | 14 | Firmicutes; Staphylococcus succinus; OTU518471 | 0.04 | 0.00 | 3 | 0.503 |
| Firmicutes; Staphylococcus; OTU356733* | 38.77 | 32.41 | 44 | Actinobacteria; Corynebacterium; OTU264597 | 1.63 | 0.20 | 26 | -0.507 |
| Firmicutes; Staphylococcus hominis; OTU154509 | 21.51 | 13.47 | 42 | Firmicutes; Staphylococcus; OTU330679* | 5.24 | 3.87 | 40 | -0.526 |
| Actinobacteria; Propionibacterium acnes; OTU368907 | 12.49 | 0.55 | 35 | Actinobacteria; Corynebacterium; OTU423737 | 0.46 | 0.00 | 16 | -0.535 |
| Actinobacteria; Propionibacterium acnes; OTU368907 | 12.49 | 0.55 | 35 | Actinobacteria; Corynebacterium; OTU264597 | 1.63 | 0.20 | 26 | -0.553 |
| Actinobacteria; Propionibacterium acnes; OTU368907 | 12.49 | 0.55 | 35 | Actinobacteria; Corynebacterium tuberculostearicum; OTU470219 | 8.74 | 5.83 | 41 | -0.560 |
| Firmicutes; Staphylococcus; OTU356733* | 38.77 | 32.41 | 44 | Firmicutes; Staphylococcus hominis; OTU338191 | 0.18 | 0.10 | 25 | -0.608 |
| Firmicutes; Staphylococcus hominis; OTU154509 | 21.51 | 13.47 | 42 | Actinobacteria; Propionibacterium acnes; OTU368907 | 12.49 | 0.55 | 35 | -0.624 |
| Actinobacteria; Propionibacterium acnes; OTU368907 | 12.49 | 0.55 | 35 | Firmicutes; Staphylococcus hominis; OTU173469 | 2.64 | 1.36 | 33 | -0.647 |
| Firmicutes; Staphylococcus; OTU356733* | 38.77 | 32.41 | 44 | Firmicutes; Staphylococcus hominis; OTU173469 | 2.64 | 1.36 | 33 | -0.652 |
| Actinobacteria; Propionibacterium acnes; OTU368907 | 12.49 | 0.55 | 35 | Firmicutes; Staphylococcus hominis; OTU338191 | 0.18 | 0.10 | 25 | -0.670 |
| Firmicutes; Staphylococcus; OTU356733* | 38.77 | 32.41 | 44 | Firmicutes; Staphylococcus hominis; OTU154509 | 21.51 | 13.47 | 42 | -0.703 |
| Proteobacteria; *Acinetobacter* | 0.04 | 0.00 | 10 | Bacteroidetes; *Prevotella* | 0.26 | 0.00 | 5 | 0.707 |
| Firmicutes; *Dialister* | 0.12 | 0.00 | 8 | Firmicutes; *Finegoldia* | 0.29 | 0.00 | 17 | 0.690 |
| Firmicutes; *Anaerococcus* | 2.02 | 0.55 | 31 | Actinobacteria; *Corynebacterium* | 13.16 | 7.39 | 41 | 0.622 |
| Firmicutes; *Finegoldia* | 0.29 | 0.00 | 17 | Actinobacteria; *Kocuria* | 0.05 | 0.00 | 9 | 0.618 |
| Firmicutes; *Peptoniphilus* | 0.35 | 0.00 | 16 | Bacteroidetes; *Porphyromonas* | 0.15 | 0.00 | 4 | 0.569 |
| Proteobacteria; *Acinetobacter* | 0.04 | 0.00 | 10 | Proteobacteria; *Roseomonas* | 0.12 | 0.00 | 7 | 0.550 |
| Firmicutes; *Finegoldia* | 0.29 | 0.00 | 17 | Bacteroidetes; *Prevotella* | 0.26 | 0.00 | 5 | 0.517 |
| Actinobacteria; *Corynebacterium* | 13.16 | 7.39 | 41 | Actinobacteria; *Propionibacterium* | 13.46 | 1.76 | 39 | -0.523 |
| Actinobacteria; *Propionibacterium* | 13.46 | 1.76 | 39 | Firmicutes; *Staphylococcus* | 69.38 | 72.31 | 44 | -0.525 |
| Actinobacteria | 26.85 | 24.22 | 44 | Firmicutes | 72.23 | 75.38 | 44 | -0.991 |

Taxa found in at least 10 samples were analysed. Only correlations (between Taxon I and Taxon II) with Sperman *r* greater than 0.5 or lower than -0.5 are presented.

* Using the naïve Bayesian classifier and the reference Greengenes taxonomy database with the confidence score threshold of 80%, the majority of sequence reads of a given *Staphylococcus* OTU could be assigned to a species as follows: OTU356733 to *S*. *epidermidis* (99.6% of sequence reads), OTU330679 to *S*. *epidermidis* (99.7% of sequence reads), OTU269541 to *S*. *epidermidis* (96.8% of sequence reads) and OTU217912 to *S*. *haemolyticus* (85.7% of sequence reads).
