# Supplementary material for: Mapping axillary microbiota responsible for body odours using a culture-independent approach
Source: Microbiome. 2015 Jan 24;3:3. doi: 10.1186/s40168-014-0064-3 (PMC4316401; doi:10.1186/s40168-014-0064-3)
Supplement: Additional file 14: Table S9. — Comparison of taxa abundance between selected female non-antiperspirant users. [file 40168_2014_64_MOESM14_ESM.docx]

**Table S9. Comparison of taxa abundance between selected female non-antiperspirant users.**

| **Chemical, olfactory and microbiological characteristics** | | **F2** | **F26** | **Change  F2 vs. F26 (%)** | **F5** | **F26** | **Change  F5 vs. F26 (%)** | **F2** | **F1** | **Change  F2 vs. F1 (%)** |
| --- | --- | --- | --- | --- | --- | --- | --- | --- | --- | --- |
| pH (median) | | 6.1 | 5.7 |  | 6.2 | 5.7 |  | 6.1 | 7.6 |  |
| Precursor | 1 | 53 | 47 | 13 | 4 | 47 | -91 | 53 | 125 | -58 |
|  | 2 | 453 | 367 | 23 | 49 | 367 | -87 | 453 | 950 | -52 |
|  | 3 | 1.036 | 2.140 | -52 | 0.090 | 2.140 | -96 | 1.036 | 2.815 | -63 |
|  | 4 | 3.144 | 3.949 | -20 | 0.389 | 3.949 | -90 | 3.144 | 8.777 | -64 |
| Odour intensity | Sulfury-cat urine | 3.128 | 0.160 |  | 3.350 | 0.160 |  | 3.128 | 1.543 |  |
|  | Fatty | 0.745 | 0.449 |  | 0.890 | 0.449 |  | 0.745 | 0.661 |  |
|  | Acid-spicy | 0.958 | 0.149 |  | 0.645 | 0.149 |  | 0.958 | 0.099 |  |
|  | Fresh onion | 3.885 | 0.198 |  | 1.190 | 0.198 |  | 3.885 | 0.115 |  |
|  | Global | 5.318 | 1.380 |  | 4.929 | 1.380 |  | 5.318 | 2.375 |  |
| Relative abundance of OTUs | *Staphylococcus* OTU356733^a^  (Firmicutes) | 26.1 | 27.5 | -5 | 51.9 | 27.5 | 89 | 26.1 | 83.4 | -69 |
|  | *Staphylococcus* *hominis* OTU154509 (Firmicutes) | 55.1 | 0.2 | 27325 | 0.5 | 0.2 | 125 | 55.1 | 0.3 | 18183 |
|  | *Propionibacterium* *acnes* OTU368907 (Actinobacteria) | 0.0 | 70.8 | -100 | 15.9 | 70.8 | -78 | 0.0 | 0.5 | -100 |
|  | *Corynebacterium* *tuberculostearicum* OTU470219 (Actinobacteria) | 9.9 | 0.0 | +^b^ | 11.1 | 0.0 | + | 9.9 | 0.4 | 2363 |
|  | *Staphylococcus* OTU330679^a^  (Firmicutes) | 0.3 | 0.0 | + | 8.6 | 0.0 | + | 0.3 | 14.5 | -98 |
|  | *Staphylococcus* *hominis* OTU173469 (Firmicutes) | 8.2 | 0.0 | + | 0.1 | 0.0 | + | 8.2 | 0.1 | 16200 |
|  | *Finegoldia* OTU86757  (Firmicutes) | 0.0 | 0.0 |  | 1.3 | 0.0 | + | 0.0 | 0.1 | -100 |
|  | *Prevotella* OTU2196  (Bacteroidetes) | 0.0 | 0.0 |  | 2.8 | 0.0 | + | 0.0 | 0.0 | + |
|  | *Roseomonas mucosa* OTU26143 (Proteobacteria) | 0.0 | 0.0 |  | 1.3 | 0.0 | + | 0.0 | 0.0 | + |
| Relative abundance of genera | *Corynebacterium* | 10.3 | 0.0 | + | 12 | 0.0 | + | 10.3 | 0.5 | 1940 |
|  | *Finegoldia* | 0.0 | 0.0 |  | 1.3 | 0.0 | + | 0.0 | 0.1 | -100 |
|  | *Prevotella* | 0.0 | 0.0 |  | 2.8 | 0.0 | + | 0.0 | 0.0 |  |
|  | *Propionibacterium* | 0.0 | 71.3 | -100 | 16 | 71 | -78 | 0.0 | 0.8 | -100 |
|  | *Roseomonas* | 0.0 | 0.1 | -100 | 1.4 | 0.1 | 2600 | 0.0 | 0.0 |  |
|  | *Staphylococcus* | 89.6 | 27.9 | 221 | 62 | 28 | 123 | 89.6 | 98.2 | -9 |
| Relative abundance of phyla | Actinobacteria | 10.3 | 71.6 | -86 | 31.2 | 71.6 | -56 | 10.3 | 1.3 | 685 |
|  | Bacteroidetes | 0.0 | 0.0 |  | 2.8 | 0.0 | + | 0.0 | 0.1 | -100 |
|  | Firmicutes | 89.6 | 27.9 | 221 | 63.9 | 27.9 | 129 | 89.6 | 98.6 | -9 |
|  | Proteobacteria | 0.1 | 0.5 | -90 | 2.6 | 0.5 | 420 | 0.1 | 0.1 | 0 |

Taxa with median relative abundance >1% in at least one subject are presented. Median values of the four sessions were calculated for pH (left axilla), odour intensity (right axilla) and relative abundance of taxa (right axilla).

^a^ Using the naïve Bayesian classifier and the reference Greengenes taxonomy database with the confidence score threshold of 80%, the majority (>99%) of sequence reads of these *Staphylococcus* OTUs were assigned to *S*. *epidermidis*.

^b^ The symbol 'plus' indicates taxa present only in the subject with a relatively high odour (F2 or F5) compared with the reference subject with a relatively low odour (F26 or F1).
