# Supplementary material for: Mapping axillary microbiota responsible for body odours using a culture-independent approach
Source: Microbiome. 2015 Jan 24;3:3. doi: 10.1186/s40168-014-0064-3 (PMC4316401; doi:10.1186/s40168-014-0064-3)
Supplement: Additional file 15: Table S10. — Sequences of eight-base barcodes for 454 pyrosequencing. [file 40168_2014_64_MOESM15_ESM.docx]

**Table S10. Sequences of eight-base barcodes for 454 pyrosequencing.**

| **Barcode** | **Subject_Session** |
| --- | --- |
| AACCGCAT | F1_1 |
| AACCGCTA | F1_2 |
| AACCGGAA | F1_3 |
| AACCTTGG | F1_4 |
| AACCTACG | F2_1 |
| AACCTAGC | F2_2 |
| AACCTTCC | F2_3 |
| AACCGGTT | F2_4 |
| AACGAACG | F3_1 |
| AACGAAGC | F3_2 |
| AACGATCC | F3_3 |
| AACGATGG | F3_4 |
| AACGCCAT | F5_1 |
| AACGCCTA | F5_2 |
| AACGCGAA | F5_3 |
| AACGCGTT | F5_4 |
| AACGTTGC | F7_2 |
| AAGCAAGC | F7_4 |
| AAGCATCC | M8_1 |
| AAGCATGG | M8_2 |
| AAGCCGAA | M8_3 |
| AAGCCGTT | M8_4 |
| AAGCGCAA | M9_1 |
| AAGCGCTT | M9_2 |
| AAGCGGAT | M9_3 |
| AAGCGGTA | M9_4 |
| AAGGAACC | M_11_1 |
| AAGGAAGG | M_11_2 |
| AAGGATCG | M_11_3 |
| AAGGATGC | M_11_4 |
| AAGGCCAA | M_12_1 |
| AAGGCCTT | M_12_2 |
| AAGGCGTA | M_12_4 |
| AATACCGC | F_14_1 |
| AATACGCC | F_14_2 |
| AATAGCGG | F_14_3 |
| AATAGGCG | F_14_4 |
| ACACCTGA | F_19_2 |
| ACACGAGA | F_19_4 |
| ACACGTCA | F_20_1 |
| ACACGTGT | F_20_2 |
| ACACTCAG | F_20_3 |
| ACACTCTC | F_20_4 |
| ACACTGAC | M_21_1 |
| ACACTGTG | M_21_2 |
| ACAGACAG | M_21_3 |
| ACAGACTC | M_21_4 |
| ACAGTGAG | M_25_1 |
| ACAGTGTC | M_25_2 |
| ACCAACCA | M_25_3 |
| ACCAACGT | M_25_4 |
| ACCAAGCT | F_26_1 |
| ACCAAGGA | F_26_2 |
| ACCACAAC | F_26_3 |
| ACCACATG | F_26_4 |
| ACCAAGGA | CTRL_1^b^ |
| ACCACAAC | CTRL_2 ^b^ |
| ACCACATG | CTRL_3 ^b^ |
| ACCACTAG | CTRL_4 ^b^ |

^a^ Sessions: 1, morning session on day 1; 2, afternoon session on day 1; 3, morning session on day 2; 4, afternoon session on day 2.

^b^ Control samples were sequences in a separate run.
